# Green synthesis of CaO-Fe₃O₄ composites for photocatalytic degradation and adsorption of synthetic dyes

**DOI:** 10.1007/s11356-025-36310-w

**Published:** 2025-03-31

**Authors:** Odín Reyes-Vallejo, Rocío Magdalena Sánchez-Albores, José Escorcia-García, Abumale Cruz-Salomón, Pascual Bartolo-Pérez, Ashok Adhikari, Maritza del Carmen Hernández-Cruz, Héctor Hiram Torres-Ventura, Héctor Armando Esquinca-Avilés

**Affiliations:** 1 Sección de Electrónica del Estado Sólido-Ingeniería Eléctrica (SEES), CINVESTAV- IPN, San Pedro Zacatenco, 07360 Mexico City, Mexico; 2 Escuela de Ciencias Químicas, Universidad Autónoma de Chiapas (UNACH), Ocozocoautla de Espinosa 29140, Chiapas, Mexico; 3 CONAHCYT-CINVESTAV del IPN, Unidad Saltillo, Ciudad de Ramos Arizpe 25900, Coahuila, Mexico; 4 Departamento de Física Aplicada, Centro de Investigación y de Estudios Avanzados del Instituto Politécnico Nacional-Unidad Mérida, Merida, 97310 Yucatán, Mexico; 5 Departamento de Materia Condensada, Instituto de Física-UNAM, Coyoacán, 04510 Mexico City, Mexico

**Keywords:** Waste reuse, Eco-friendly synthesis, Photocatalysis, Adsorption, Composite material, Synthetic dyes

## Abstract

The efficient removal of synthetic dyes, such as methylene blue (MB) and malachite green (MG), continues to pose a significant challenge due to their high stability, toxicity, and resistance to conventional treatment methods. In this study, CaO-Fe₃O₄ compounds were synthesized using a sustainable ball-milling technique, utilizing calcium oxide derived from eggshells and Fe₃O₄. The compounds were calcined at temperatures ranging from 200 to 800 °C to optimize their structural and photocatalytic properties. The sample calcined at 400 °C exhibited the highest surface area (17.86 m^2^/g), the narrowest bandgap (2.10 eV), and the coexistence of CaO, Ca(OH)₂, and γ-Fe₂O₃ phases, making it an ideal candidate for achieving high dye removal efficiency. Under visible light, this sample completely degraded MB at 10 ppm within 30 min, following pseudo-first-order kinetics with a rate constant (*k*ₐₚₚ) of 0.110 min^−1^ and a half-life (*t*₁_/_₂) of 6.30 min. At an MB concentration of 50 ppm, complete degradation was achieved in 90 min. Radical scavenging experiments indicated that superoxide radicals (·O₂^−^) played a key role in the degradation mechanism. For MG (100 ppm), the maximum adsorption capacity (*q*ₑ) was 1111.11 mg/g, fitting the Langmuir model (*R*^2^ = 0.996) with an equilibrium constant (*K*_L_) of 0.6822 L/mg, indicating a highly favorable process. The adsorption kinetics followed a pseudo-second-order model (*R*^2^ ≈ 0.999), suggesting chemisorption as the rate-limiting step. Thermodynamic parameters confirmed that MG adsorption was spontaneous and endothermic, with negative Gibbs free energy, positive enthalpy, and increased entropy. This study proposes an eco-friendly and efficient approach for dye removal, integrating waste valorization.

**Graphical Abstract:**

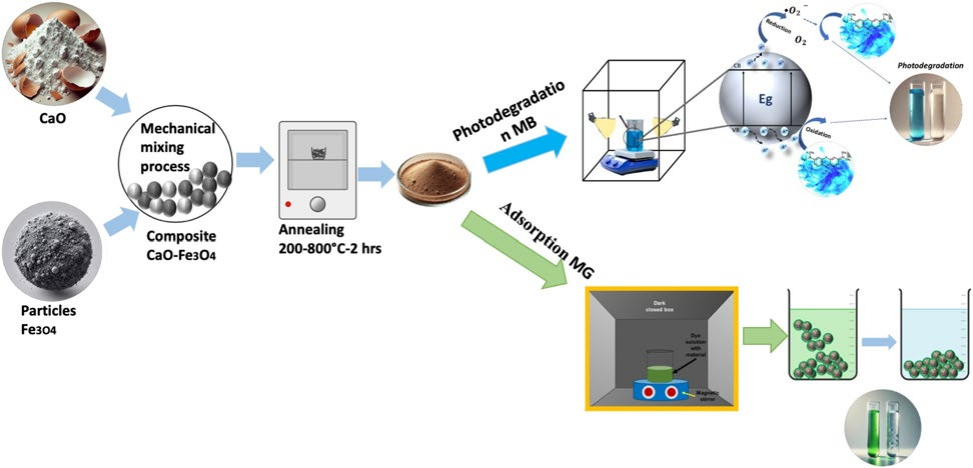

**Supplementary Information:**

The online version contains supplementary material available at 10.1007/s11356-025-36310-w.

## Introduction

The contamination of water by synthetic dyes, such as methylene blue (MB) and malachite green (MG), represents a significant environmental challenge. MB is a thiazine dye widely used in the textile, leather, and paper industries, among others, which is non-biodegradable and difficult to degrade due to its aromatic benzene structure (Rahmi and Lelifajri 2017). Its release into wastewater without proper treatment contributes to water pollution and poses health risks, including skin irritation, gastrointestinal issues, and mutagenic and carcinogenic effects. Similarly, MG is a highly toxic dye used in various industries and is classified as carcinogenic, mutagenic, and teratogenic. These dyes disrupt water-based ecosystems by hindering photosynthesis in aquatic plants, threatening biodiversity, and causing severe health issues, including immune system damage and harm to vital organs (Aziz et al. 2024). These pollutants are highly toxic and resistant to conventional treatment methods, significantly affecting water quality and public health (Crini and Lichtfouse 2019; Raouf et al. 2019). This concern is further exacerbated in the context of the United Nations Sustainable Development Goals (SDGs), which emphasize the importance of improving water management to achieve global targets in health, environmental sustainability, and circular economy. Wastewater treatment emerges as a pivotal approach, capable of contributing to more than 50% of the 17 SDGs (Delanka-Pedige et al. 2021).

Advanced oxidation processes (AOPs), particularly photocatalysis, stand out as excellent alternatives for removing organic contaminants due to their simplicity, affordability, high efficiency, and ecological compatibility. This process employs semiconductor materials activated by light absorption, generating hole-electron pairs that interact with water and dissolved oxygen, producing hydroxyl (·OH) and superoxide (·O₂^−^) radicals, which drive the degradation of contaminants. Photocatalysis has proven its effectiveness in treating dyes such as MB, MG, rhodamine B (RB), and methyl orange (MO), as well as in the photoreduction of heavy metals like chromium or arsenic (Durán-Álvarez et al. 2024; Sánchez-Albores et al. 2020; Tahmasebi et al. 2019). These attributes make photocatalysis a promising approach for addressing the complex challenges posed by various wastewater pollutants while contributing to the SDGs related to water quality and environmental health. Despite these advantages, significant limitations remain. Traditional photocatalytic materials, such as TiO_2_ and ZnO, primarily absorb UV light, limiting their efficiency under visible light (Sun et al. 2023). However, its synthesis processes often involve unsustainable methods that do not adhere to the principles of green chemistry that encompass physical, chemical, and biological approaches with their own characteristics in terms of sustainability, efficiency, and applicability. This is why green synthesis has gained relevance due to its ability to use agro-industrial waste, such as fruit peels, eggshells, stems, and leaves, as reducing and stabilizing agents, thereby promoting an eco-friendly and low-cost strategy. In comparison, physical methods such as thermal evaporation and sputtering allow for the production of materials with high purity and structural control, but they require specialized equipment and high energy consumption. Chemical methods, such as the sol–gel process, offer versatility in obtaining nanoparticles with adjustable properties, although they often involve the use of chemical precursors and solvents that can generate hazardous waste (Rathod et al. 2024). On the other hand, ball milling emerges as an efficient and sustainable alternative, as it does not require additional chemical reagents and allows for the synthesis of nanoparticles from solid precursor materials through the application of controlled mechanical forces. Unlike other methods, ball milling enables the processing of a wide variety of materials, including oxides and metals, offering precise control over particle size distribution without the need for extreme temperature or pressure conditions. This combination of accessibility, scalability, and compatibility with various applications makes it a key technique in the development of sustainable nanomaterials. Therefore, the development of materials that efficiently absorb visible light and incorporate waste reutilization strategies presents a dual challenge for advancing wastewater treatment technologies.

In this context, organic waste materials are utilized for the synthesis of functional materials, offering an innovative and sustainable solution. A prominent example of byproducts generated in large quantities is eggshells. Besides being a rich source of nutrients for humans, eggs have a global consumption exceeding one million metric tons per year, leading to the generation of significant amounts of waste (Vanthana Sree et al. 2020). These eggshells, primarily composed of calcium carbonate (CaCO₃), can be transformed into calcium oxide (CaO) through simple and sustainable synthesis processes aligned with the principles of green chemistry. This approach not only valorizes waste materials but also produces a material with unique properties for photocatalysis and adsorption applications.

A promising strategy to enhance the photocatalytic properties of CaO is its combination with smaller bandgap materials, such as magnetite (Fe_3_O_4_). These iron oxides expand light absorption into the visible spectrum and provide suitable magnetic properties for facilitating material recovery and reuse. Previous studies have demonstrated the potential of combining oxides like CaO and Fe₃O₄ in various catalytic applications, such as biodiesel production, where CaO acts as a basic catalyst, and Fe₃O₄ serves as a magnetic support (Aleman-Ramirez et al. 2023). However, the specific combination of CaO-Fe₃O₄ for photocatalytic and adsorption applications has not been extensively investigated. Therefore, there is a need for innovative opportunities to develop a material that efficiently degrades organic pollutants under visible light and adsorbs substances to maximize its functionality.

In addition to photocatalytic processes, adsorption has emerged as a promising and eco-friendly method for removing contaminants, particularly those that are difficult to degrade, as it generates non-toxic by-products (Cano et al. 2025). Several studies have explored the removal of dyes through adsorption, such as the work by Shi et al., who developed carbon/zeolite composite materials (CZCM). These materials exhibited an adsorption capacity (*q*ₑ) of up to 9705 mg/g and a removal efficiency of 97.05% for malachite green dye. The mesoporous structure of these materials, along with the presence of metallic elements such as iron (Fe) and calcium (Ca), as well as functional groups like carboxyl and hydroxyl, significantly enhanced their adsorption performance (Shi et al. 2025).

Similarly, Zhu et al. synthesized a nanocomposite of iron oxide (γ-Fe₂O₃) dispersed on reduced graphene oxide (rGO) sheets using a sonication process. By employing response surface methodology (RSM), they determined that under optimal conditions (a dose of 200 mg/100 mL, a pH of 7.99, and a contact time of 112.68 min), a removal efficiency of approximately 90% was achieved, with an adsorption capacity (*q*ₑ) of 40.64 mg/g. Further optimization of the adsorption parameters resulted in a removal efficiency of 98% and an improved adsorption capacity of 64.26 mg/g, demonstrating the effectiveness of the nanocomposite in contaminant removal (Zhu et al. 2025).

While activated carbon is widely recognized as an effective adsorbent due to its exceptional adsorption capacity, its high production costs pose a significant limitation. Despite its commercial use, the search for alternative, cost-effective adsorbents is a priority in the field of water treatment. Waste-derived materials, such as agricultural by-products, have gained attention as cost-effective alternatives to address these drawbacks. For instance, agricultural waste-derived macadamia nutshell activated carbon (MAC) demonstrates a high surface area and has proven effective for phenol and triclosan adsorption (Yimrattanabovorn et al. 2024). Similarly, utilizing waste materials such as eggshells as a calcium source for photocatalytic applications represents a sustainable and cost-effective approach. These materials are also combined with others for adsorption and advanced treatment methods, further enhancing their effectiveness. Such innovations highlight the potential of adsorption-based processes as practical solutions for pollutant removal, aligning with sustainability goals while lowering production costs (Al-mahmodi et al. 2025; Mergbi et al. 2023; Munir et al. 2025).

Iron-based materials like magnetite (Fe_3_O_4_) and maghemite (γ-Fe_2_O_3_) have demonstrated strong adsorption activity for dyes such as Congo red (CR), MB, and MG (Afkhami et al. 2010; W. Li et al. 2021). These materials provide magnetic properties for easy recovery, enhanced light absorption due to reduced bandgaps, and increased contaminant affinity through adsorption. Furthermore, they remain chemically stable during photocatalytic and adsorption processes, making them versatile candidates for wastewater treatment. Ecological considerations are paramount in developing semiconductor materials. Minimizing the excessive use of harmful chemicals, energy consumption, and waste materials is considered according to the strategies of green chemistry (Zuliani and Cova 2021). One of the best examples is reusing waste materials (e.g., eggshells), which is a promising approach to assigning value to discarded materials while preventing their disposal. Sustainable techniques, such as biosynthesis using biomass extracts, microwave-assisted processes, and ball milling, are gaining recognition for their ability to significantly reduce energy consumption and promote environmental sustainability. These methods align with key sustainability principles by minimizing material and energy use, reducing waste production, and fostering faster and safer processes for operators. Moreover, these green technologies contribute to more efficient and environmentally friendly production, aligning with the growing demand for sustainable industrial practices (Li et al. 2025; Pattanayak et al. 2025). Materials derived from waste, including animal bones, fruit shells, and eggshells, have shown significant promise in a variety of applications, particularly in the fields of catalysis, energy storage, and water treatment. These materials not only offer a sustainable alternative to conventional resources but also contribute to reducing environmental impact by recycling waste into valuable products with multifunctional properties (Rasheed et al. 2025; Reyes-Vallejo, et al. 2024a; Tan et al. 2025).

This study focuses on the synthesis of CaO-Fe₃O₄ compounds using the ball milling technique, with eggshells as a calcium source. This compound combines the advantages of the high reactivity of CaO and the magnetic properties of Fe₃O₄, making it ideal for photocatalytic applications. These characteristics enable efficient recovery and reuse of the photocatalyst, offering significant economic advantages for large-scale applications. Recent research has highlighted the limitations of conventional adsorbents and photocatalysts, such as limited adsorption capacity, low stability, difficulty in recycling, and high costs (Satyam and Patra 2024)**.** Although materials like carbon-based compounds, metal oxides, and polymers have been widely explored, many of these materials require energy-intensive synthesis processes and exhibit low recovery potential, limiting their practical applicability (AlAqad et al. 2025). On the other hand, mechanical ball milling emerges as a cost-effective, scalable, and environmentally friendly alternative for synthesizing this catalyst (Ali et al. 2025). This technique allows for homogeneous mixing of precursors, avoids using toxic reagents, and operates under ambient conditions, reducing energy consumption compared to traditional chemical or high-temperature synthesis methods. Additionally, ball milling improves particle dispersion and surface activation, which can enhance the catalytic and adsorptive properties of the final material.

A sustainable and efficient strategy is proposed for developing CaO-Fe₃O₄ compounds with enhanced dye removal capabilities, aligning with the principles of green chemistry and the circular economy. A thorough literature review revealed that the specific combination of CaO and Fe₃O₄ could be a viable option for synthesizing these compounds. To date, the use of CaO-Fe₃O₄ as a catalyst for the removal of methylene blue and malachite green had not been reported. This study addresses this gap by proposing a novel approach that integrates a sustainable synthesis method with improved photocatalytic and adsorptive properties, contributing to the development of new materials for wastewater treatment. By leveraging the synergy between CaO and Fe₃O₄, this study aims to develop a material that not only achieves high contaminant removal efficiencies but also facilitates easy recovery due to its magnetic properties. In this work, the structural and functional properties of the compound were analyzed to optimize its efficiency in the photocatalytic degradation of MB and the adsorption of MG. These findings underscore the potential of the CaO-Fe₃O₄ compound as a cost-effective, sustainable, and scalable solution for wastewater treatment.

## Materials and methods

**Fig. 1:**
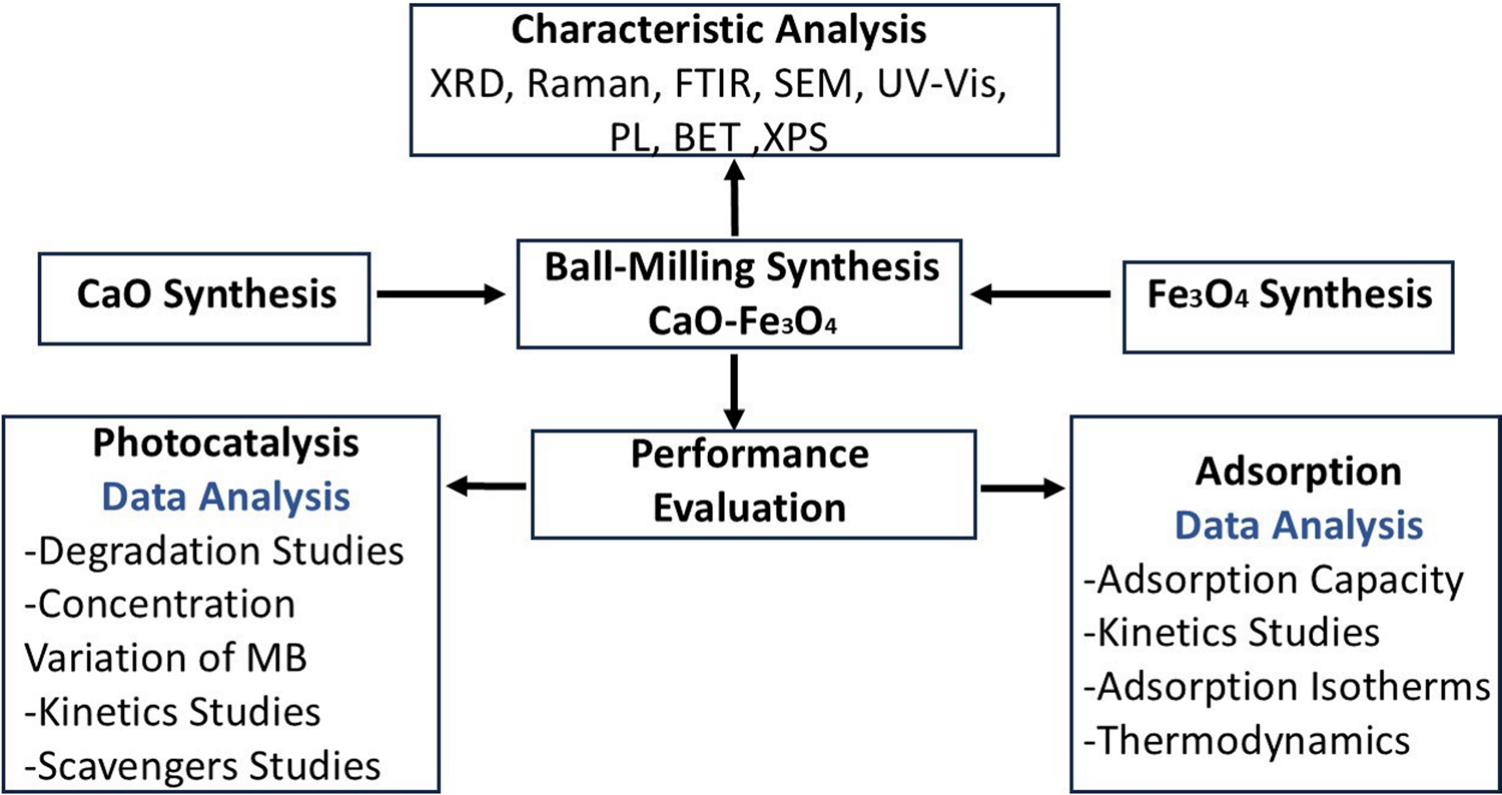
Schematic representation of the methodology used in the study

A study framework was designed to evaluate the synthesis, characterization, and application of CaO-Fe₃O₄ materials for photocatalysis and adsorption processes, as outlined in Fig. 1. The methodology involved synthesizing the materials, characterizing their properties using advanced analytical techniques, and assessing their performance through degradation and adsorption experiments. Each step of the framework is described in detail in the following sections.

### Chemicals

Methylene blue (C_16_H_18_C_l_N_3_S·3H_2_O, CAS: 61–73-4, Fig. 2a), which has a water solubility of 40 g L^−1^, and malachite green (C_48_H_50_N_4_O_4_·2HC_2_O_4_, CAS: 569–64-2, Fig. 2b), with a water solubility of 20 g L^−1^, were purchased from Sigma-Aldrich-Merck, Darmstadt, Germany.

**Fig. 2:**
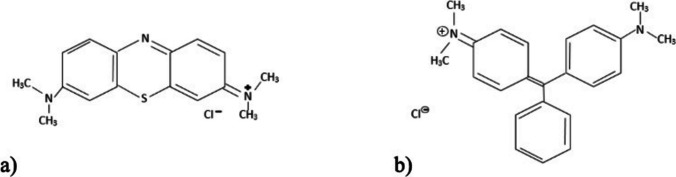
Chemical structures of the dyes: **a** methylene blue and **b** malachite green

### CaO synthesis using eggshells

Eggshells obtained from a Mexican public market were thoroughly cleaned with water and then dried at 60 °C for 24 h in a conventional laboratory oven, model MED-H-3. Subsequently, the dry eggshells were crushed using a standard blender and then sieved through a mesh with a 0.75-mm opening to ensure uniformity and standardize particle size. The obtained particles were subjected to several washes, approximately four times, with hot water at around 60 °C. After completing the washing process, they were dried in an oven at 60 °C for 24 h and placed on a tray to ensure uniform drying. The resulting eggshell powder was annealed at 900 °C in a furnace under atmospheric air for 5 h to produce CaO (Reyes-Vallejo et al. 2023).

### Fe_3_O_4_ synthesis

A solution of iron salts was prepared by dissolving 4.50 g of FeSO₄·7H₂O (22.5 mmol) and 7.65 g of Fe₂(SO₄)₃ (22.5 mmol) in a 1:1 molar ratio in 100 mL of deionized water. The dissolution process was carried out on a hot stirring plate, using a magnetic stirrer set to a medium stirring speed to ensure the complete dissolution of the salts. Once the iron salts were fully dissolved, concentrated NH₄OH was added dropwise to adjust the pH of the solution to 9.5. This step was performed carefully to prevent a rapid increase in pH. The mixture was then heated to 80 °C in a thermostatic bath and maintained at this temperature for approximately 30 min under constant stirring until black particles precipitated. At this point, a magnet was employed to separate the magnetic particles from the solution. The obtained black particles were washed with ethanol and deionized water several times to remove soluble impurities. After washing, the particles were dried in an oven at 70 °C for 6 h to ensure the complete removal of any residual moisture.

### CaO-Fe_3_O_4_ composite synthesis

**Fig. 3:**
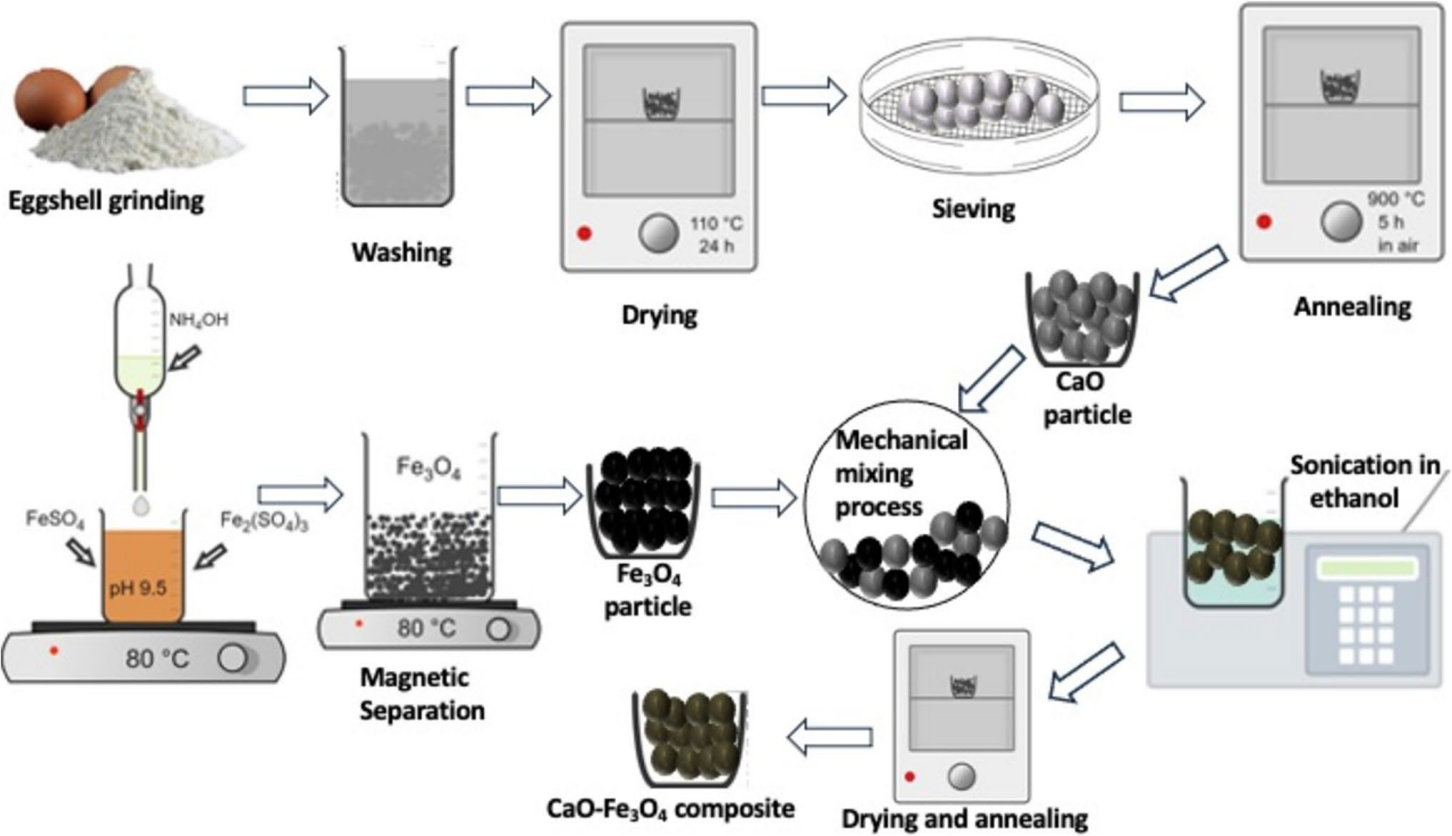
The CaO-Fe_3_O_4_ composite synthesis process

The CaO-Fe₃O₄ composite was synthesized by weighing 1 g of Fe₃O₄ and 19 g of CaO. The materials were thoroughly mixed via ball milling using ten tungsten carbide balls at 400 rpm for 3 h under dry conditions to ensure uniform particle distribution and close physical contact between the components. The resulting powder was then combined with 30 mL of ethanol to form a slurry, which was subjected to ultrasonic processing at a frequency of 40 kHz for 1 h under constant stirring to enhance particle dispersion and interaction. Following the ultrasonic treatment, the slurry was dried overnight at 100 °C to remove residual ethanol. The dried composite was then calcined by heating at a controlled rate of 10 °C/min for 2 h across a temperature range of 200 to 800 °C, facilitating the formation of the CaO-Fe₃O₄ composite (Aleman-Ramirez et al. 2023). The synthesis process is depicted in Fig. 3.

### Characterization

The structures of the prepared samples were investigated using powder X-ray diffraction (XRD). A D2 PHASER Bruker diffractometer, equipped with Cu-Kα radiation (λ = 1.54056 Å), was used in a 2θ range from 10° to 70° and operated at a scan rate of 2°/min. Raman spectroscopy characterization was performed using an NTEGRA SPECTRA NT-MDT system, utilizing a 532-nm laser emission as the excitation source. The surface functional groups of the samples were investigated using Fourier transform infrared spectroscopy (FTIR). The FTIR spectrum was obtained with a Nicolet™ 6700 spectrometer (Thermo Fisher Scientific, Massachusetts, USA) in attenuated total reflectance (ATR) mode, covering a range from 525 to 4000 cm^−1^. The spectra were recorded with 52 scans, a resolution of 32, a sample gain of 1.0, a mirror speed of 0.4747, and an aperture of 80. The surface morphology of the prepared samples was studied by the field emission scanning electron microscopy with a Hitachi FE-SEM S-5500. Absorbance was measured using a JASCO UV–Vis spectrophotometer (model V-670), and diffuse reflectance was recorded with an integrating sphere attached to the same instrument. Photoluminescence (PL) emission spectra were acquired using a BWTEK Exemplar spectrometer with a 365-nm UV LED as the excitation source.

The crystallite size (*D*) was estimated using the Scherrer equation (Eq. 1) based on the XRD data.

1 $$D=\frac{K\times \lambda }{{\beta }_{hkl\,\times\, COS\theta }}$$

Where *K* is the shape factor, which can range from 0.62 to 2.08; however, its exact value for this material system is unknown. For this study, *K* value was taken as 0.9 (Hassanzadeh-Tabrizi 2023). The parameter *λ* represents the wavelength of Cu-K *α* radiation (*λ* = 1.54056 Å), ${\beta }_{hkl}$ is the peak width at half maximum intensity of the plane (*hkl*), and *θ* is the Bragg’s angle of the plane (*hkl*). Therefore, the crystallite sizes reported for all samples are estimations based on these parameters.

The bandgap energy (*E*_g_) was determined by plotting ${(F\left(R\right)\text{h}v)}^{2}$ vs $hv$ and estimating *E*_g_ from the intersection of the linear fit with the *x*-axis. The bandgap was estimated using Eq. 2.

2 $${(F\left(R\right)\text{h}v)}^{2}=\text{A}\left(hv-{E}_{g}\right)$$

3 $$F\left(R\right)=\frac{{(1-R)}^{2}}{2R}$$

where *F*(*R*) is the Kubelka–Munk function (Eq. 3) calculated using the diffuse reflectance (*R*), $hv$ is the photon energy, and *A* is a constant (Kubelka 1931).

The specific surface area and pore volume of the samples were assessed through nitrogen physisorption employing a surface area and pore size analyzer (Micromeritics VacPrep 061). Approximately 0.1 g of the material was degassed for 24 h at 120 °C and 6.6 kPa of pressure. The surface area was determined using the Brunauer–Emmett–Teller (BET) technique after introducing nitrogen gas and following the degassing process (Brunauer et al. 1938). Additionally, the micropore volume of the samples was calculated using the t-Plot method (Barrett et al. 1951), and X-ray photoelectron spectrometry (XPS) analysis was performed on the samples using a Thermo Scientific ESCALAB 250Xi device to determine the elemental composition and chemical states of the surface.

### Dyes removal processes

#### Photocatalysis of methylene blue

It is described in two different ways:

- a) The photocatalytic test was conducted as follows: MB solutions with concentrations ranging from 10 to 50 ppm were prepared in 150-mL beakers. Then, 50 mg of the catalyst was added to each solution. All tests were performed with constant stirring at a speed of 550 rpm using a magnetic stirrer. Initially, the MB solution was stirred in the dark for 30 min to achieve adsorption–desorption equilibrium between the catalyst and the solution. Subsequently, two 100-W LED lamps emitting visible light were turned on, placed 15 cm above the solution.

Once the lamps were activated, 3-mL aliquots were collected at 5-min intervals throughout the experiment. These aliquots were then centrifuged at 4000 rpm for 3 min to remove any excess catalyst. The absorbance of the centrifuged aliquots was measured at a wavelength of 663 nm to determine the remaining MB concentration in each sample.

The MB degradation percentage was estimated using Eq. 4 (Santis et al. 2024)**.**

4 $$\%\, \text{Degradation}=\frac{{C}_{\text{O}}-{C}_{\text{f}}}{{C}_{\text{O}}}\times 100$$

where *C*_0_ and *C*_f_ are the initial and final concentrations of MB, respectively.

The reaction kinetics of the photocatalytic MB removal were analyzed employing the pseudo-first-order kinetic model, given by Eq. 5, where *C*_0_ is the initial concentration, *k* is the apparent kinetic constant (min^−1^) estimated from the slope of the linear fitting, and* C* is the measured concentration at time *t* (min). The half-life period (*t*_1/2_) of MB in the photocatalytic system was calculated using Eq. 6 (Li et al. 2014).

5 $$\text{ln}\left(\frac{{C}_{0}}{C}\right)=kt$$

6 $${t}_{1/2}=\frac{\text{ln}(2)}{{K}_{\text{app}}}$$

Furthermore, various tests were conducted to confirm that MB removal is not exclusively due to photocatalysis but also to rule out contributions from other processes, such as photolysis or adsorption, as discussed further.

- b) The photolysis test was conducted to determine whether the MB dye could degrade solely due to the presence of light. This test was performed under the same conditions as the photocatalytic test described in section a, but without the catalyst, using only the dye and the LED lamp light.

The adsorption test was carried out as follows: a 100-mL solution containing 10 ppm of MB and 50 mg of the composite was used without light. The solution was vigorously stirred at 550 rpm in the dark for 30 min. After that, 3-mL aliquots were withdrawn every 5 min, then centrifuged at 4000 rpm for 3 min. The absorbance was measured at 663 nm using a UV–Vis spectrophotometer (Thermo Scientific GENESYS 10S) to determine the concentration of methylene blue removed solely by adsorption.

Additionally, trapping studies using scavengers were conducted to elucidate the nature of the reactive species involved in the photocatalytic process. Radical scavengers, namely benzoquinone (BQ), ethylenediaminetetraacetic acid (EDTA), and isopropyl alcohol (IPA), were introduced individually into the MB solution. These scavengers were utilized at concentrations of 0.001 mol L^−1^ each and acted as quenchers for specific reactive species: BQ for ·O_2_^−^, EDTA for holes (h^+^), and IPA for ·OH.

#### Adsorption of malachite green

The adsorption test was carried out as follows: a 100-mL solution containing 100 ppm of MG and 10 mg of composite was used. The solution was vigorously stirred at 550 rpm in the dark for 45 min. Later, 3-mL aliquots from the solution were taken every 3 min and centrifuged immediately at 5500 rpm, and their absorbance was measured at 617 nm using a UV–Vis spectrophotometer (Thermo Scientific GENESYS 10S).

The adsorption capacity (*q*_e_, mg/g) was determined at equilibrium using Eq. 7, where *C*_0_ and *C*_e_ represent the initial and equilibrium concentrations (mg/L), respectively; *V* is the volume of the solution used (L); and *M* is the mass of the adsorbent (g) (Crini et al. 2007). These parameters provide a comprehensive understanding of the adsorption efficiency and capacity of the synthesized photocatalyst for the removal of MG.

7 $${q}_{e}=\left[\frac{{C}_{\text{o}}-{C}_{\text{e}}}{M} \right]\times 100$$

The MG removal efficiency (η) was calculated (see Eq. 8) by comparing the initial dye concentration (*C*_0_) before treatment with the final concentration (*C*_*t*_) at a specific time (*t*) during the adsorption tests.

8 $$\eta (\%)=\left[\frac{{C}_{\text{o}}-{C}_{t}}{{C}_{0}}\right]\times 100$$

## Results and discussion

### CaO-Fe_3_O_4_ composite characterization

**Fig. 4:**
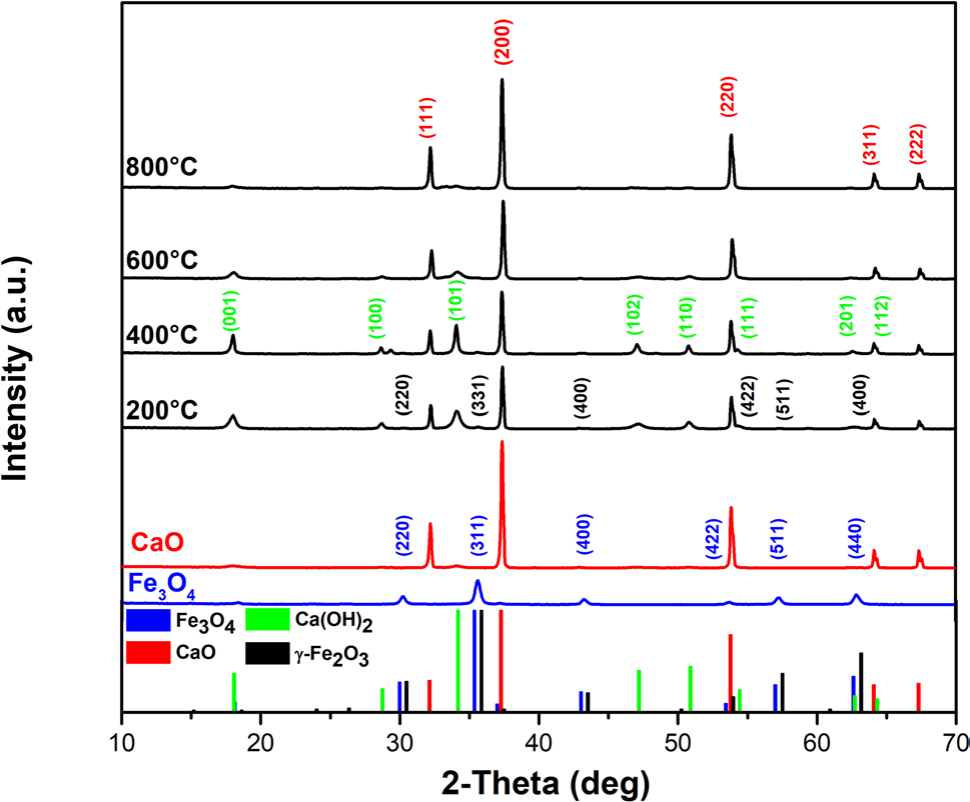
XRD patterns of Fe_3_O_4_, CaO, and composites annealed from 200 to 800 °C

The XRD patterns depicted in Fig. 4 reveal distinct features for Fe_3_O_4_, CaO, and the annealed composites. The XRD results exhibit characteristic diffraction peaks corresponding to the Fe_3_O_4_ phase (Magnetite PDF 01–071–6337) at the (220), (311), (400), (422), (511), and (450) planes (Bakr et al. 2020), while the (111), (200), (220), (222), and (311) planes are attributed to CaO (Lime PDF 37–1497) as observed in another published research (Habte et al. 2019). The (101) plane indicates a slight presence of Ca(OH)₂ (Portlandite, PDF 44–1481) in the CaO, which is attributed to moisture adsorbed during cooling after annealing. In the composite, this phase is significantly increased due to the reaction between CaO and ethanol during mechanical mixing and drying. As Ca(OH)₂ oxidizes to CaO, its presence decreases with rising annealing temperatures. However, not all Ca(OH)₂ transforms into CaO at 800 °C. Given that Fe₃O₄ is chemically unstable above 200 °C (Ounacer et al. 2020), the annealing process promotes its oxidation to γ-Fe₂O₃ (maghemite, PDF 39–1346). This phenomenon has been extensively documented in the literature, where it has been demonstrated that heating Fe₃O₄ to temperatures of approximately 300 °C induces its transformation into γ-Fe₂O₃, as observed in previous studies on the thermal evaluation of magnetite (Aliahmad and Nasiri Moghaddam 2013). Notably, the peaks corresponding to the iron oxide phases are small due to their low content in the composite (5%). Furthermore, annealing treatment enhanced the crystallinity and crystallite size of the composites while reducing their surface area (see Table 1). This suggests that annealing might affect the contact between the composite and contaminants, potentially limiting adsorption and dye removal efficiency (Reyes-Vallejo et al. 2023).

**Table 1:** Structural, surface, and optical parameters of annealed CaO-Fe_3_O_4_ composite samples

Annealing temperature (°C)	Crystallite size *D* (nm)	Surface area (m^2^/g)	Bandgap (eV)	Particle size—SEM (nm)
*200*	*37*	*13.40*	*2.15*	*22–26*
400	42	17.86	2.10	12–18
600	49	9.83	2.14	24–28
800	51	7.35	2.15	85–100

**Fig. 5:**
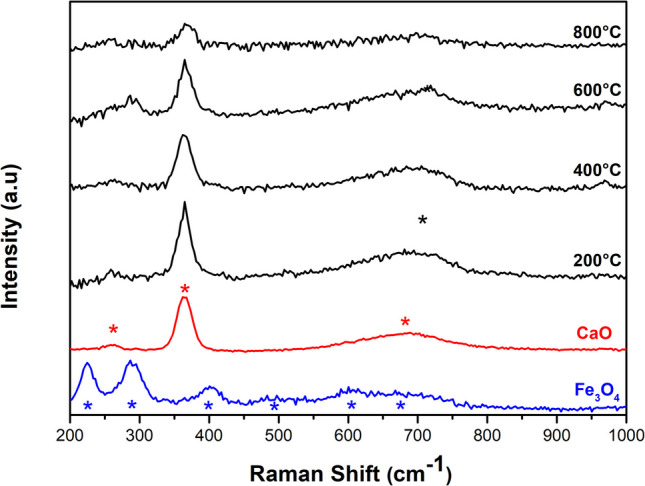
Raman spectra of Fe_3_O_4_, CaO, and composites annealed from 200 to 800 °C

Figure 5 displays the Raman spectra of the samples. Peaks associated with Fe_3_O_4_ (* blue) are observed at 399, 496, 606, and 676 cm^−1^, corresponding to the Fe–O vibration mode (Mishra and Ramaprabhu 2011; Shebanova and Lazor 2003; Yew et al. 2017; Yuvakkumar and Hong 2014). The oxidation of Fe_3_O_4_ introduces additional peaks at 225 and 288 cm^−1^ (Yew et al. 2017). In the CaO sample (* red), Raman peaks found at 259, 365, and 678 cm^−1^ are attributed to Ca-O bonds (Galvan-Ruiz et al. 2007; Jaiswal et al. 2021). These peaks are also linked to the Ca(OH)_2_ structure (Schmida and Dariz 2014), making it challenging to attribute them to a single component. Instead, they result from contributions by both CaO and Ca(OH)_2_. Significant changes occur upon mixing and annealing processes. At 200 °C, the peak at 365 cm^−1^ attains the highest intensity, indicating a substantial Ca(OH)_2_ content (Ingole et al. 2017), which is consistent with XRD measurements. As the temperature increases further, the intensity of the 365 cm^−1^ peak diminishes, signifying the transformation of Ca(OH)_2_ into the CaO phase (Ingole et al. 2017). Moreover, the composites do not exhibit peaks corresponding to the Fe_3_O_4_ phase. However, the broadening of the peak at 678 cm^−1^ (* black) may indicate the presence of the maghemite phase, suggesting the oxidation of magnetite to maghemite during the annealing process (Chernyshova et al. 2007; Jubb and Allen 2010).

**Fig. 6:**
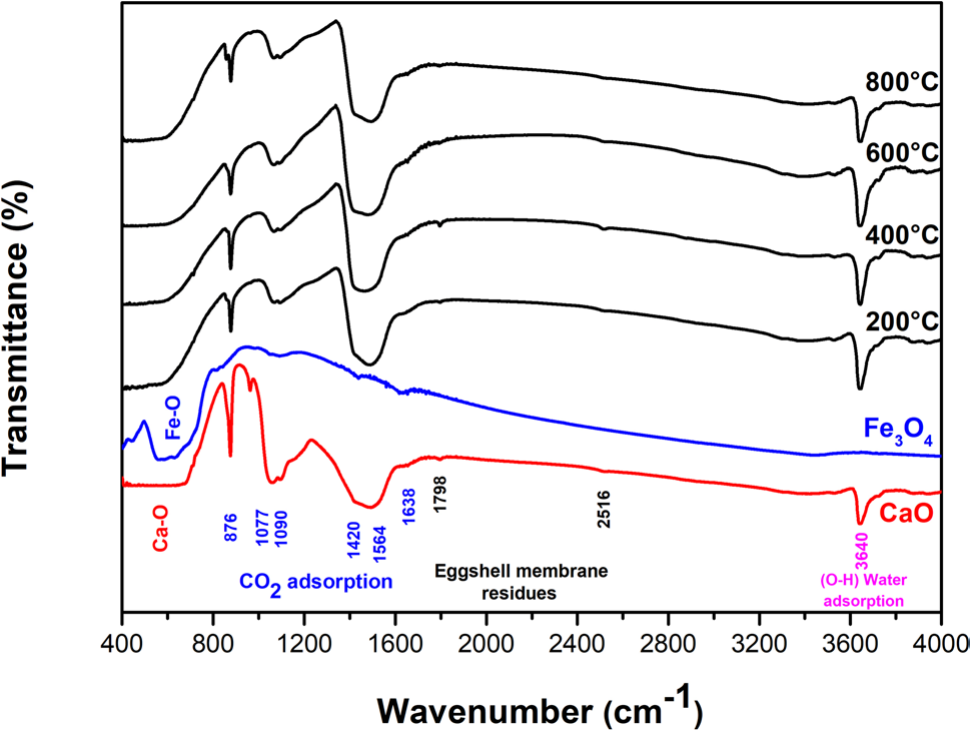
FTIR spectra of Fe_3_O_4_, CaO, and composites annealed from 200 to 800 °C

The FTIR spectra of samples annealed at different temperatures are shown in Fig. 6. The peak at 445 cm^−1^ and a broad peak between 500 and 800 cm^−1^ are attributed to Fe–O bonds of Fe_3_O_4_ (Mishra and Ramaprabhu 2011; Nalbandian et al. 2015; Yuvakkumar and Hong 2014). Furthermore, the peak at 3425 cm^−1^ is associated with water adsorption (Jaiswal et al. 2021). Regarding the CaO sample and the CaO-Fe_3_O_4_ composites, similar spectra are observed, likely due to the high content of the CaO precursor (95%). The Ca-O bonds are observed in the range of 500 to 700 cm^−1^, suggesting strong absorption in this region (Galvan-Ruiz et al. 2007; Jaiswal et al. 2021). The peaks at 876, 1077, 1090, 1420, 1564, and 1638 cm^−1^ are attributed to several C-O bonds and occasionally to the presence of a carbonate group (Carvalho et al. 2011). The absence of bands around 2900 and 1850 cm^−1^, typically associated with the C = O carbonate group, indicates the minimal presence of the carbonate phase (Putkham et al. 2022). Additionally, the XRD and Raman analyses did not detect any CaCO₃, suggesting the presence of CO and CO₂, which may result from adsorption during the cooling process after annealing and from the interaction of organic residues with ethanol (Galvan-Ruiz et al. 2007; Jaiswal et al. 2021). On the other hand, the strong absorption peak at 3640 cm^−1^ confirms the presence of the Ca(OH)_2_ phase related to O–H bonds (Jaiswal et al. 2021; Kodeh et al. 2020). Also, the peaks at 1798 and 2516 cm^−1^, corresponding to amine and amide residues from the eggshell membrane, were recorded (Carvalho et al. 2011; Jaiswal et al. 2021).

**Fig. 7:**
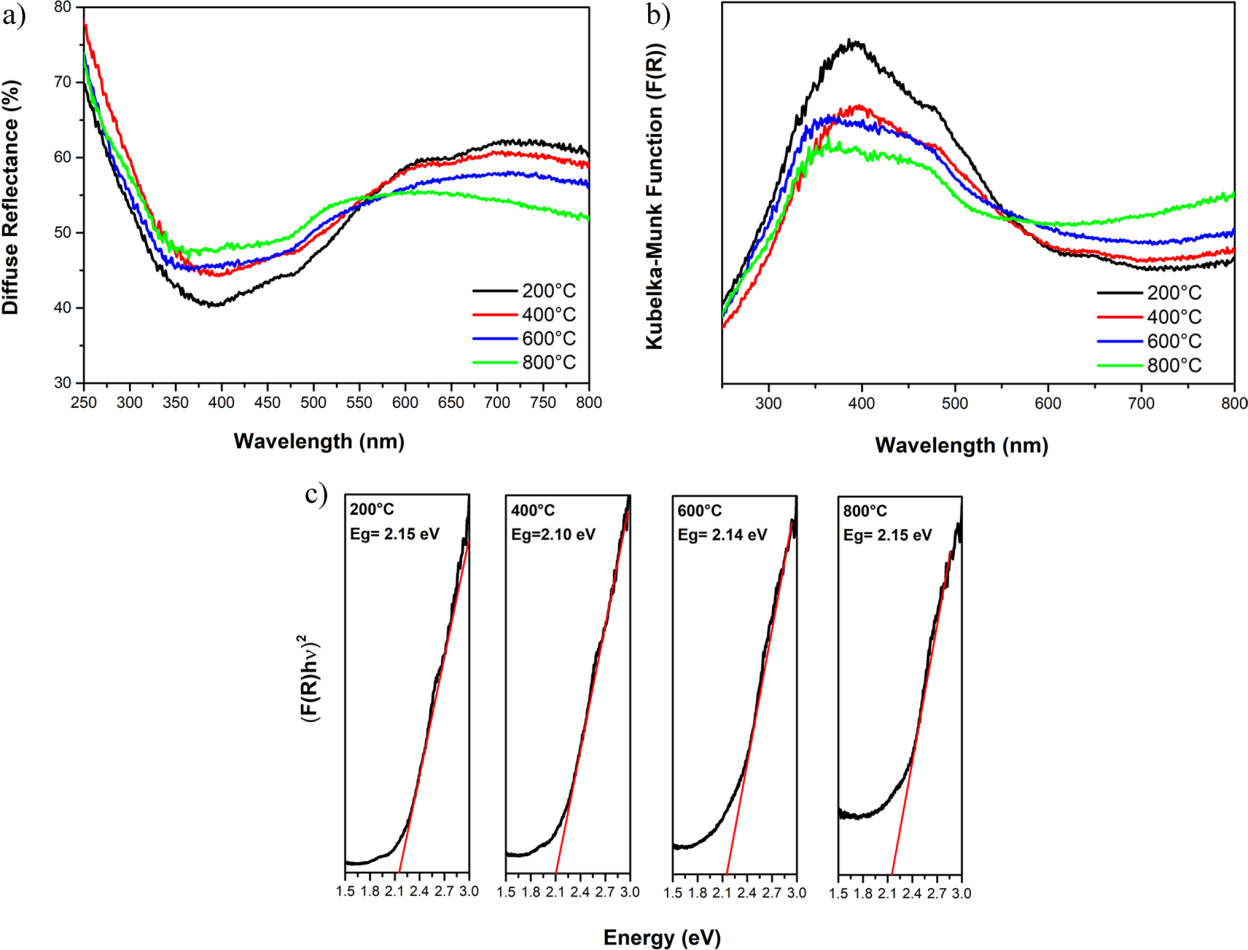
**a** Diffuse reflectance, **b** Kubelka–Munk function, and **c** bandgap estimation of composites annealed from 200 to 800 °C

The diffuse reflectance and bandgap estimation of composites annealed at different temperatures ranging from 200 to 800 °C are shown in Fig. 7. As the annealing temperature increases, a gradual rise in reflectance is observed for all samples, particularly below 570 nm in the bandgap region (see Fig. 7a). This increase corresponds to a slight decrease in the Kubelka–Munk function value, indicating a reduction in the absorption coefficient, as shown in Fig. 7b. As a result, a marginal reduction in visible light absorption capacity is observed with increasing temperature.

The *E*_g_ values derived from Kubelka–Munk plots (see Fig. 7c) exhibit minimal variation, with the lowest value of 2.10 eV found for the sample annealed at 400 °C. Typically, CaO synthesized from eggshells exhibits two bandgaps (i.e., 5.54 and 2.27 eV) depending on the CaO phases present (Jaiswal et al. 2021). In contrast, Ca(OH)₂ has an *E*_g_ of only 1.8 eV (Mathivanan et al. 2018). Additionally, Fe₃O₄ presents a bandgap of 2.0 to 2.1 eV (Manikandan et al. 2014), while maghemite exhibits *E*_g_ values of 1.77 and 1.91 eV (Silva et al. 2013).

The bandgap reduction from 2.15 to 2.10 eV as the temperature increases from 200 to 400 °C is primarily attributed to the oxidation of ferrite to maghemite. Additionally, the improved crystallinity of the Ca(OH)₂ phase may also contribute to this behavior. Subsequently, as the temperature increases from 400 to 800 °C, the bandgap rises from 2.10 to 2.15 eV, which is associated with the transformation of calcium hydroxide into calcium oxide. This trend is clearly observed in the diffractogram shown in Fig. 4.

**Fig. 8:**
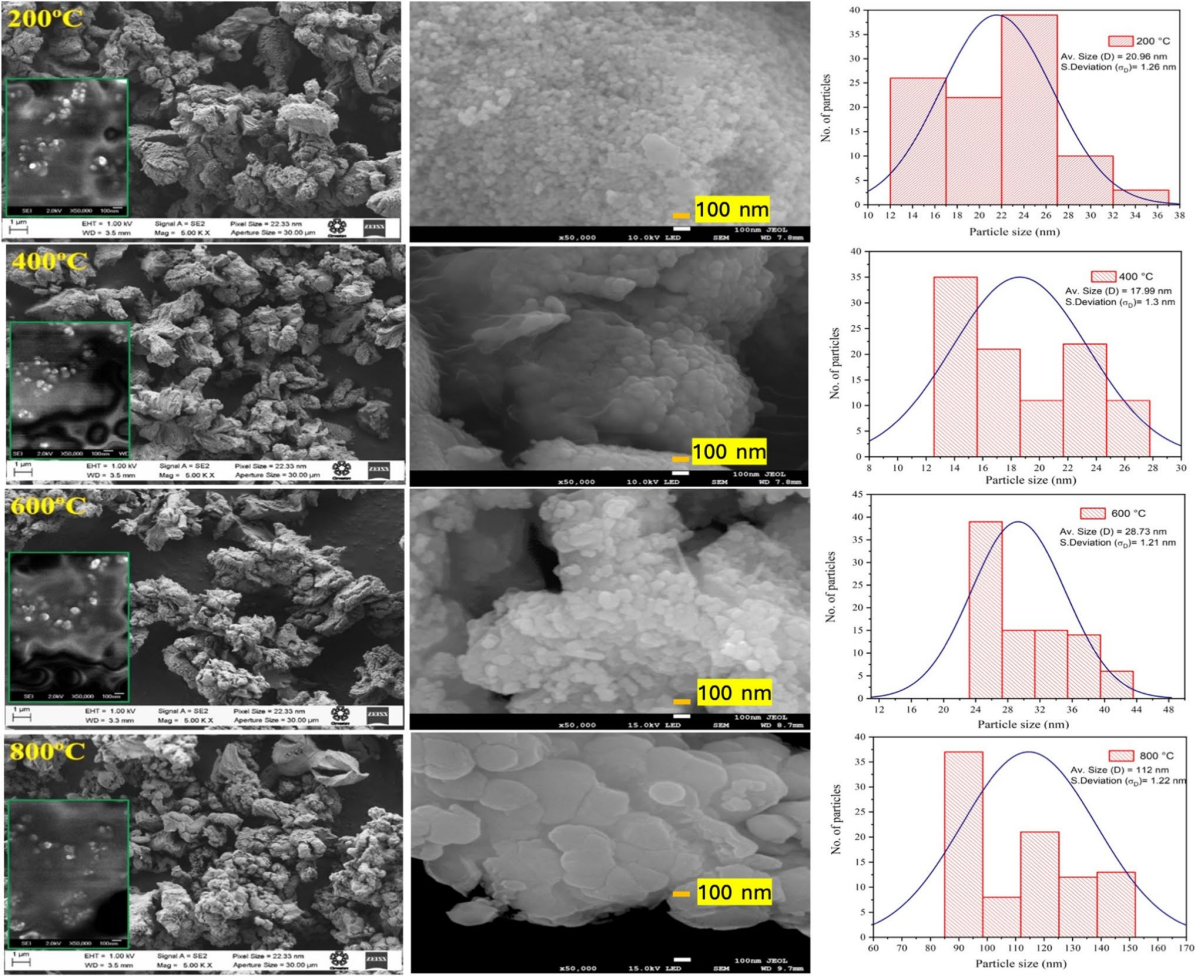
FE-SEM images of composites annealed from 200 to 800 °C

The annealed composite samples exhibit an indistinct surface morphology with agglomerated particles at various temperatures (see Fig. 8). These agglomerates vary in size and shape, with dimensions exceeding 5 μm. Size distribution analysis reveals that the particles forming the agglomerates range in size from a few to several tens of nanometers (see Fig. 8 and Table 1).

To calculate the particle size, the particles in the FE-SEM images were measured by fitting the histogram with a log–normal function, which is expressed as (Eq. 9):

9 $$f\left(D\right)=\left(\frac{1}{\sqrt{2\pi {\sigma }_{D}}}\right)exp\left[-\frac{{ln}^{2}\left(\frac{D}{{D}_{0}}\right)}{2{\sigma }^{2}}\right]$$

where *D* corresponds to the average particle size and ${\sigma }_{D}$ is the standard deviation.

As the annealing temperature increases from 200 to 400 °C, cracks begin to appear on the surface of the agglomerates, leading to an increase in surface area and a slight decrease in particle size (see Table 1). This increase in surface area is beneficial for enhancing contact with contaminants. However, when the annealing temperature exceeds 400 °C, the particle surfaces become more compact and exhibit fewer cracks, resulting in a decrease in surface area and an increase in particle size. This effect is most pronounced in the sample annealed at 800 °C, where nearly all calcium hydroxide is transformed into calcium oxide. Additionally, the oxidation of magnetite to maghemite during thermal processes introduces cationic vacancies in the crystal structure, altering the magnetic interactions between particles and leading to an increase in particle size (Jafari et al. 2015).

This transformation is attributed to atomic rearrangement, as the high energy at this temperature enhances atomic mobility, promoting a coalescence effect. In this process, smaller particles merge to form larger ones, thereby reducing surface energy (see Table 1). These observations align with the XRD analysis, further confirming the structural changes induced by varying annealing temperatures.

**Fig. 9:**
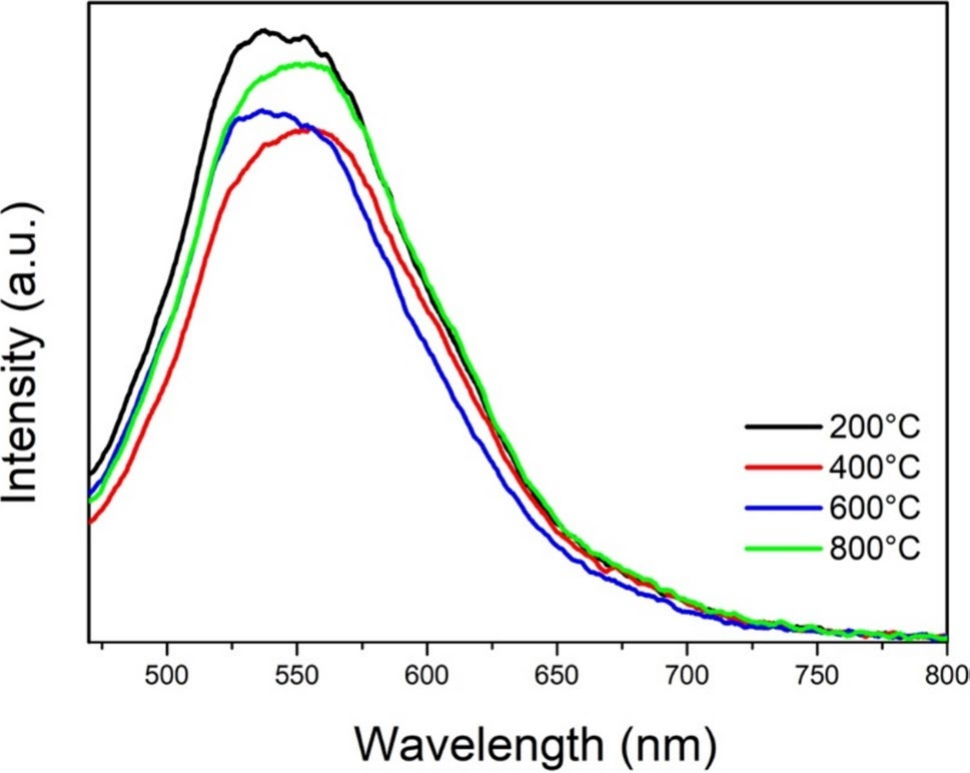
Photoluminescence spectra of composites annealed from 200 to 800 °C

Figure 9 presents the PL spectra of annealed composites. All samples exhibit a broad peak around 550 nm, which is attributed to oxygen vacancies (Coleto et al. 2020; Reyes-Vallejo et al. 2023). The composite samples annealed at temperatures of 400 and 600 °C showed the lowest peak intensity, indicating reduced radiative recombination for these samples. This reduction might be due to their improved charge transport characteristics. Conversely, the sample annealed at 200 °C shows the highest intensity peak, likely due to its lower crystallinity, containing more defect densities than other samples. The lower crystallinity makes it more difficult for this sample to transport photogenerated charges efficiently, resulting in increased recombination effects.

**Fig. 10:**
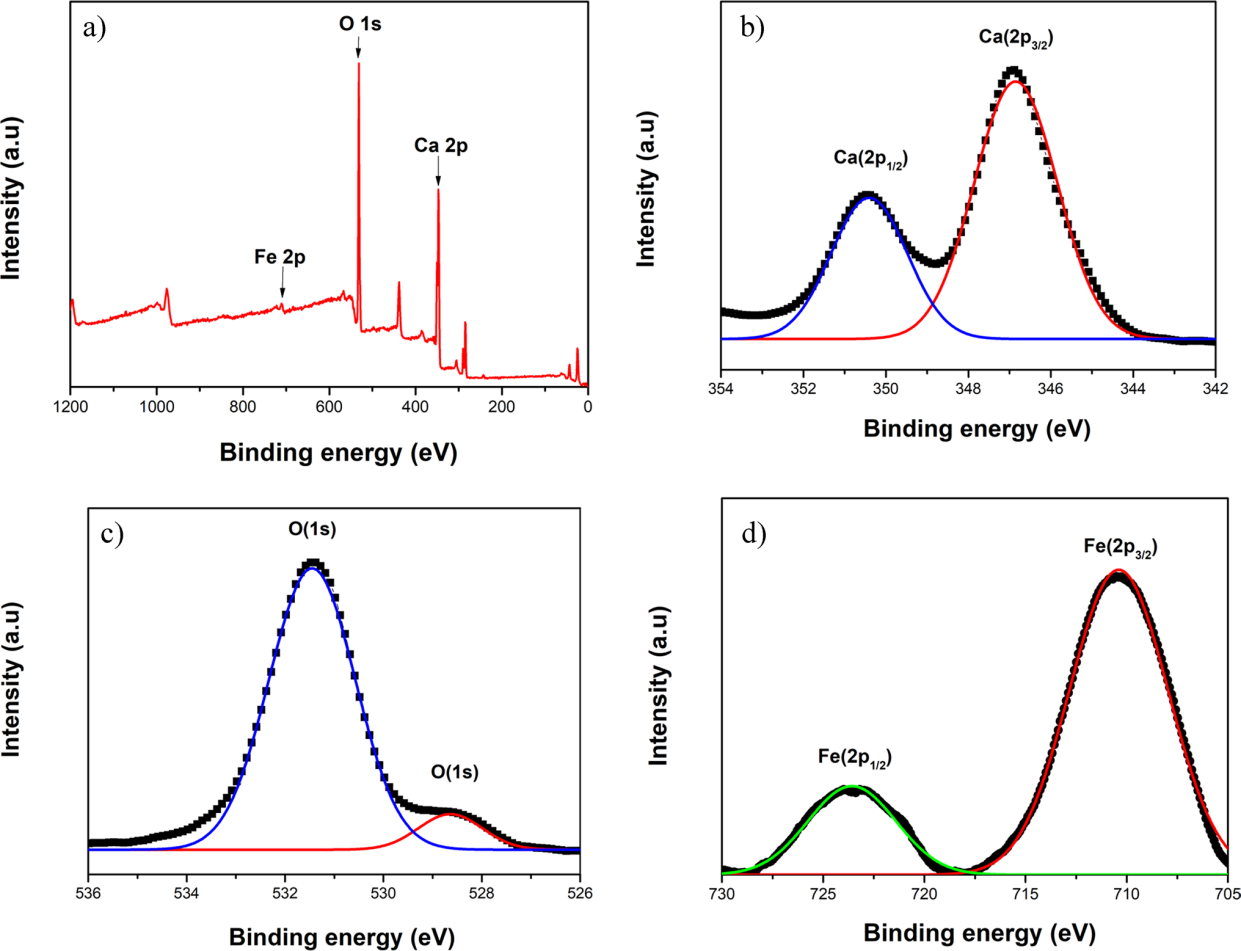
XPS spectra of CaO-Fe_3_O_4_ composite annealed at 400 °C: **a** survey, **b** Ca(2p), **c** O(1 s), and **d** Fe(2p)

Furthermore, XPS spectra of the CaO-Fe_3_O_4_ composite annealed at 400 °C are shown in Fig. 10. The survey XPS spectrum (see Fig. 10a) reveals characteristic core levels of elements constituting the compound, namely Ca 2p, O 1 s, and Fe 2p. The Ca 2p_1/2_ and Ca 2p_3/2_ binding energies were determined at 346.85 and 350.41 eV, respectively (see Fig. 10b). The energetic distance of 3.5 eV between these peaks confirms the + 2 oxidation state of calcium (Minakshi et al. 2019). In the case of the high-resolution XPS spectrum of O 1 s, two peaks were detected at 531.45 and 528.58 eV (see Fig. 10c). These peaks are associated with bonding involving inorganic elements like calcium in materials derived from eggshells (Al-Muhtaseb 2021; Minakshi et al. 2019). Finally, the Fe 2p_3/2_ and Fe 2p_1/2_ binding energies were recorded at 710.39 and 723.58 eV (see Fig. 10d), confirming the + 3 oxidation state of iron and hence the presence of the maghemite phase (Wang et al. 2013). The absence of a shoulder or signal around 709 eV provides evidence that the Fe₃O₄ phase was completely oxidized to maghemite. Additionally, the lack of satellite peaks confirms that Fe₂O₃ is present in its maghemite phase rather than hematite.

### Dye removal efficiency

#### Photocatalytic study for the methylene blue removal

**Fig. 11:**
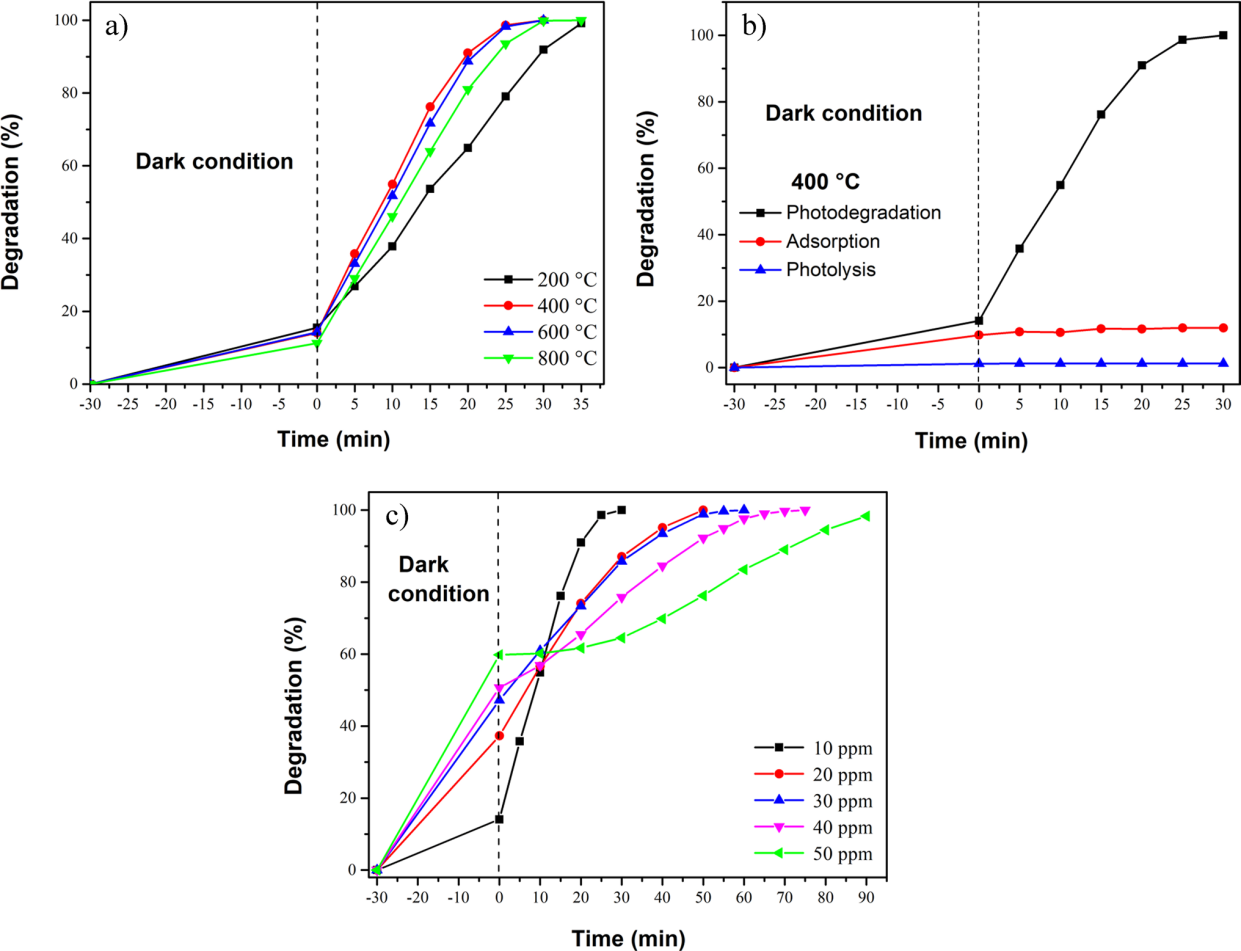
Photodegradation of MB (10 ppm) by annealed CaO-Fe_3_O_4_ composites: **a** effect of varying the temperature from 200 to 800 °C and **b** photolysis, adsorption, and photodegradation of MB by sample annealed at 400°C. **c** Effect of the MB concentration using the sample annealed at 400 °C. In all cases, 50 mg of composite and 100 mL of solution were used

The photocatalytic removal of MB with the concentration of 10 ppm using CaO-Fe₃O₄ composites annealed at temperatures ranging from 200 to 800 °C is presented in Fig. 11a. The composite annealed at 400 °C exhibited the highest efficiency in dye removal within 30 min compared to the other samples. This superior performance can be attributed to the larger surface area of the composite and its smaller band gap, which together maximize surface contact with MB and enhance light absorption. In contrast, further increasing the annealing temperature reduces the surface area and increases the band gap values, thereby limiting the efficiency of the samples annealed at 600 and 800 °C.

According to the PL results, the sample annealed at 400 °C exhibits a lower intensity signal, indicating improved charge transport. As reported in previous studies, this efficient separation of charge carriers is a key factor in enhancing photocatalytic activity, which explains the superior photodegradation performance observed at this temperature (Cao et al. 2015).

The MB adsorption behavior of the composite sample annealed at 400 °C is depicted in Fig. 11b. As shown, a near-equilibrium condition is observed throughout the test, with an MB removal of approximately 12% after the dark stage (first 30 min of agitation). This demonstrates that 30 min is sufficient to reach equilibrium conditions. The photolysis test indicated negligible MB removal, suggesting that MB removal is mainly due to photodegradation.

A significant removal of MB was achieved with only 50 mg of the composite. Here, the effect of increasing MB concentration on efficiency was studied by testing solutions ranging from 10 to 50 ppm (see Fig. 11c). It was found that MB adsorption increased with higher initial MB concentrations, likely due to enhanced contact between the dye molecules and the material. However, the photodegradation reaction slowed down as MB concentration increased, despite complete removal being achieved in all cases. This decrease in reaction rate can be attributed to two factors: (1) the higher initial dye load, which requires the degradation of a larger amount of MB, and (2) the reduced light penetration caused by the more concentrated solution. For example, in the case of 50 ppm, during the dark stage, 60% of the dye is removed, i.e., about 30 ppm, while when 10 ppm is used, about 10% is removed, i.e., 1 ppm. This means that during the photodegradation stage, in the first case, 20 ppm remains to be removed, while in the second, 9 ppm remains; hence, the photodegradation process takes considerably longer when 50 ppm is used. However, it is noteworthy that complete removal of the 50 ppm is achieved in 90 min of illumination.

**Fig. 12:**
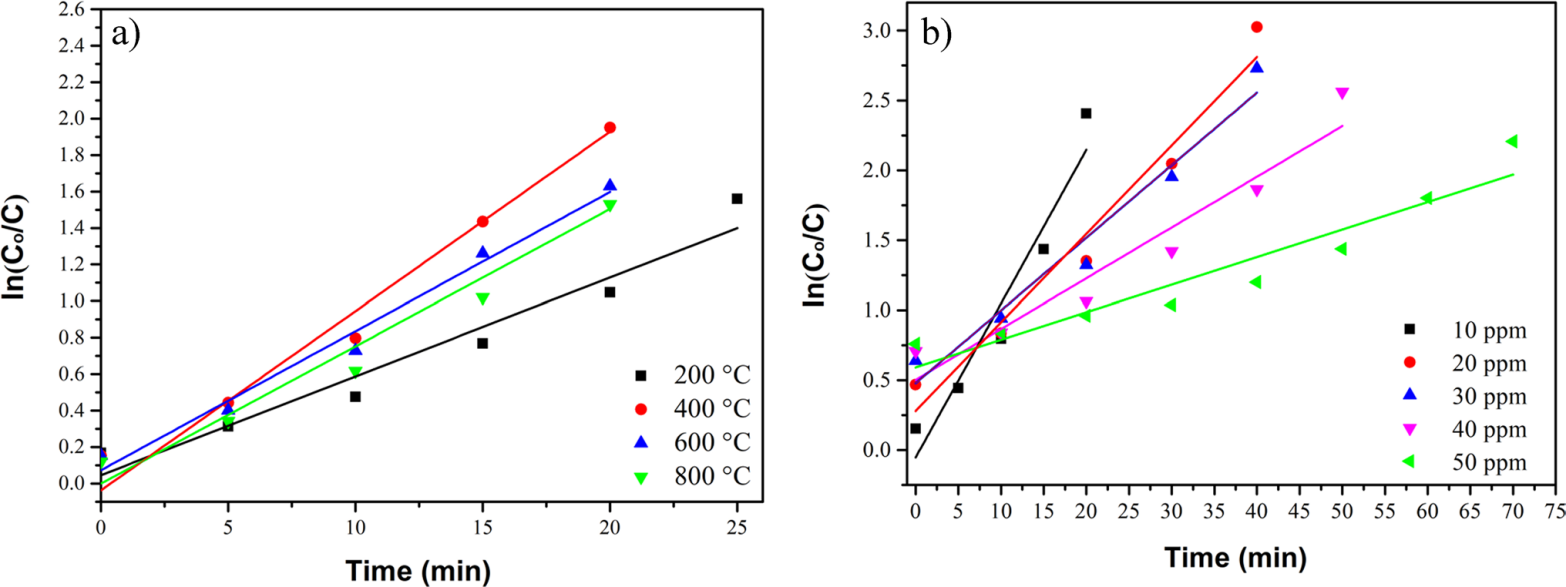
The kinetic study of MB photodegradation (10 ppm) by CaO-Fe_3_O_4_ composites: **a** effect of annealing temperature and **b** effect of the MB concentration using the sample annealed at 400 °C

Regarding the investigation of reaction kinetics, the effect of varying the annealing temperatures of the composite and the MB concentrations was studied, as presented in Fig. 12. The data fit the pseudo-first-order model in all cases, as indicated by the *R*^2^ values (see Tables 2 and 3). The composites annealed at 400 °C demonstrate the best performance, exhibiting the lowest *t*_1/2_ and the highest *k*_app_ value (see Table 2). Conversely, when MB concentration increases (see Table 3), a rise in the *t*_1/2_ values and a reduction in the *k*_app_ values are noticed, consistent with the results in Fig. 11c.

**Table 2:** Kinetic parameters of the effect of annealing temperature on CaO-Fe_3_O_4_ composites

Annealing temperature (°C)	*k*_app_ (min^−1^)	*t*_1/2_ (min)	*R*^2^ adj
200	0.054	12.79	0.936
400	0.110	6.30	0.917
600	0.098	7.05	0.907
800	0.075	9.21	0.937

**Table 3:** Kinetic parameters of the effect of MB concentration using the CaO-Fe_3_O_4_ composite annealed at 400°C

ppm	*k*_app_ (min^−1^)	*t*_1/2_ (min)	*R*^2^ adj
10	0.110	6.30	0.917
20	0.063	11.0	0.954
30	0.052	13.34	0.950
40	0.037	18.82	0.916
50	0.005	154.03	0.901

**Fig. 13:**
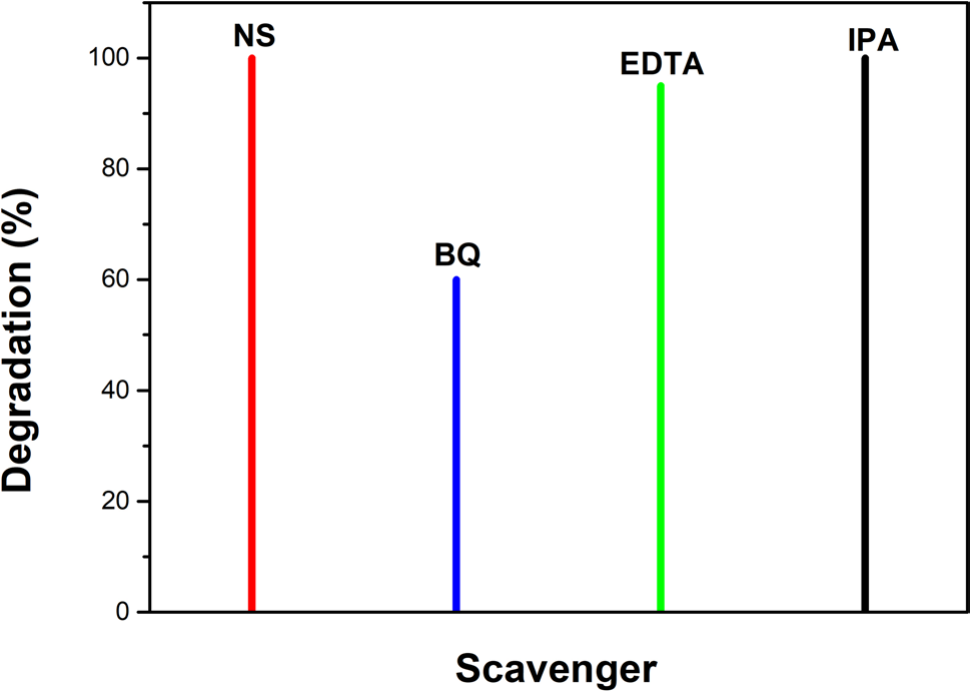
Influence of scavengers on the MB photodegradation using CaO-Fe_3_O_4_ sample annealed at 400 °C. Acronyms: NS (no scavengers), BQ (benzoquinone), EDTA (ethylenediaminetetraacetic acid), IPA (isopropanol)

The radical trapping experiment was performed to identify the reactive species involved in the MB photodegradation. The results showed that the BQ significantly inhibits the effect on MB photodegradation, indicating the crucial role of superoxide radicals (·O₂^−^) in the process. In contrast, the EDTA had a minimal effect, suggesting that holes (h^+^) play a role but are less critical. The IPA had no effect, showing no influence of hydroxyl radicals (·OH) (see Fig. 13). These results suggest that superoxide radicals are the primary reactive species driving the MB photodegradation, corroborating earlier studies that employed CaO derived from eggshells for similar applications (Vanthana Sree et al. 2020).

According to trapping experiments, the composite photodegrades MB through the processes described in Eqs. 10–14. First, the composite becomes excited when irradiated with light (*hv*), leading to the generation of electron–hole pairs. During this process, an electron (e^−^) is promoted to the higher band (i.e., conduction band) from the lower band (i.e., valence band), leaving behind a positive hole (h^+^_VB_) in the valence band (see Eq. 10) (Selvaraj et al. 2021). This step is crucial in photocatalysis since electrons and holes are reactive species that contribute to the removal of contaminants. Subsequently, the electrons in the conduction band (e^−^_CB_) can react with oxygen (O_2_) present in the solution, producing superoxide radicals (·O₂^−^) (see Eq. 11). Simultaneously, holes in the valence band (h^+^_VB_) can directly oxidize MB molecules or react with species present in the solution (Santhosh et al. 2018), such as hydroxyl ions (OH^**−**^) or water (H_2_O), generating hydroxyl radicals (·OH) (see Eqs. 12 and 13). However, in this case, it has been observed that hydroxyl radicals do not play a significant role in MB degradation since using isopropanol (IPA) as an ·OH scavenger did not show a noticeable effect on the photodegradation. Therefore, the contribution of hydroxyl radicals is minimal in this process.

The MB photodegradation is carried out mainly through the combined action of holes (h^+^_VB_) and superoxide radicals (·O_2_.^−^) that attack MB molecules, breaking their chemical bonds and transforming them into less complex and less toxic degradation products (see Eq. 14)

10 $$\text{Composite semiconductor }+ hv\to {{\text{e}}^{-}}_{\text{CB}}+{{\text{h}}^{+}}_{\text{VB}}$$

11 $${{\text{e}}^{-}}_{\text{CB}}+{\text{O}}_{2}\to \cdot {{\text{O}}_{2}}^{-}$$

12 $${\text{H}}_{2}\text{O}+{{\text{h}}^{+}}_{\text{VB}}\to {\text{H}}^{+}+\cdot \text{OH}$$

13 $${{\text{h}}^{+}}_{\text{VB}}+{\text{OH}}^{-}\to \cdot \text{OH}$$

14 $$\text{MB}+{{\text{h}}^{+}}_{\text{VB}}+\cdot {{\text{O}}_{2}}^{-}\to \text{Degradation by}-\text{products}$$

**Fig. 14:**
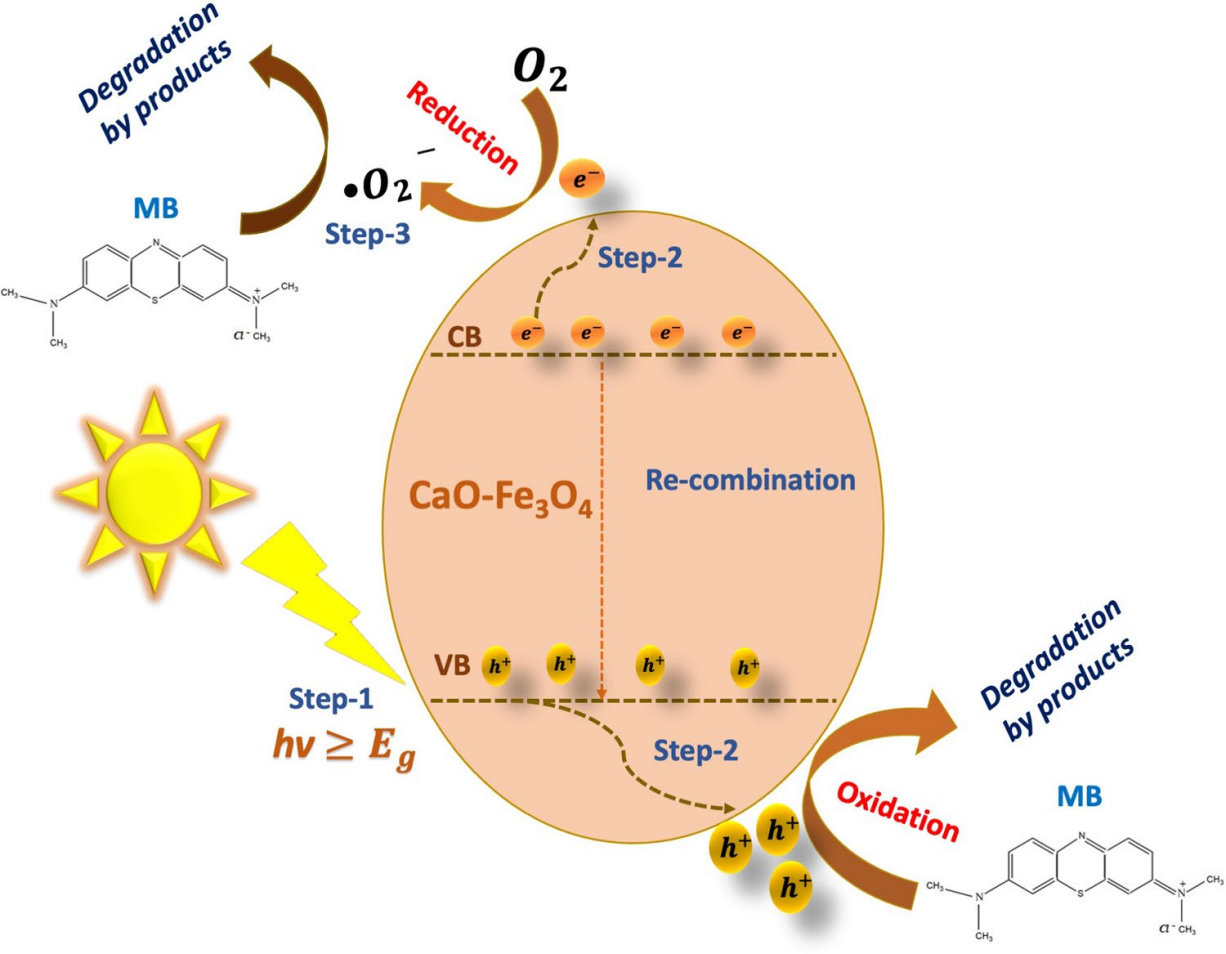
Schematic representation of the photodegradation mechanism of MB by CaO-Fe_3_O_4_ sample annealed at 400 °C

Figure 14 schematically illustrates the activation of the photocatalyst and the subsequent MB degradation. Table 4 compares MB removal efficiency through photodegradation with similar materials, demonstrating that this process is highly competitive compared to materials based on CaO or Fe_3_O_4_.

**Table 4:** MB photodegradation reports of different photocatalysts

Material	Synthesis method	Dosage catalyst (mg/mL)	Light source (W)	Dye (ppm)	Removal (%)	Time (min)	Ref
Fe_3_O_4_/SiO_2_/MnO_2_/BiOBr-Bi	Solvothermal	15/65	Metal halide lamp (400 W)	10	95.23	150	Ma et al. (2021)
Fe_3_O_4_/ZnO	Solid-state	200/50	Halogen lamp (500 W/cm^2^)	100	88.5	120	Elshypany et al. (2021)
Fe_3_O_4_/ZnWO_4_/CeVO_4_	Co-precipitation	30/300	Xenon lamp (250 W)	25	84	120	Marsooli et al. (2020)
Fe_3_O_4_/MWCNT	One-step co-precipitation method	40/100	Sodium lamp (400 W)	10	98.49	60	Hussain et al. (2022)
CaO	Eggshell annealed	12.5/100	Natural light	10	98	180	Jaiswal et al. (2021)
CaO/MgO	Ball milling/annealing	100/100	LED light (200W)	10	20	100	Reyes-Vallejo et al. (2023)
CaO/Fe_3_O_4_	Mechanical griding/annealing	10/100	LED lights (100 W)	10 20 30 40 50	100 100 100 100 100	30 50 60 75 90	**This work**

#### Adsorption study for the malachite green removal

**Fig. 15:**
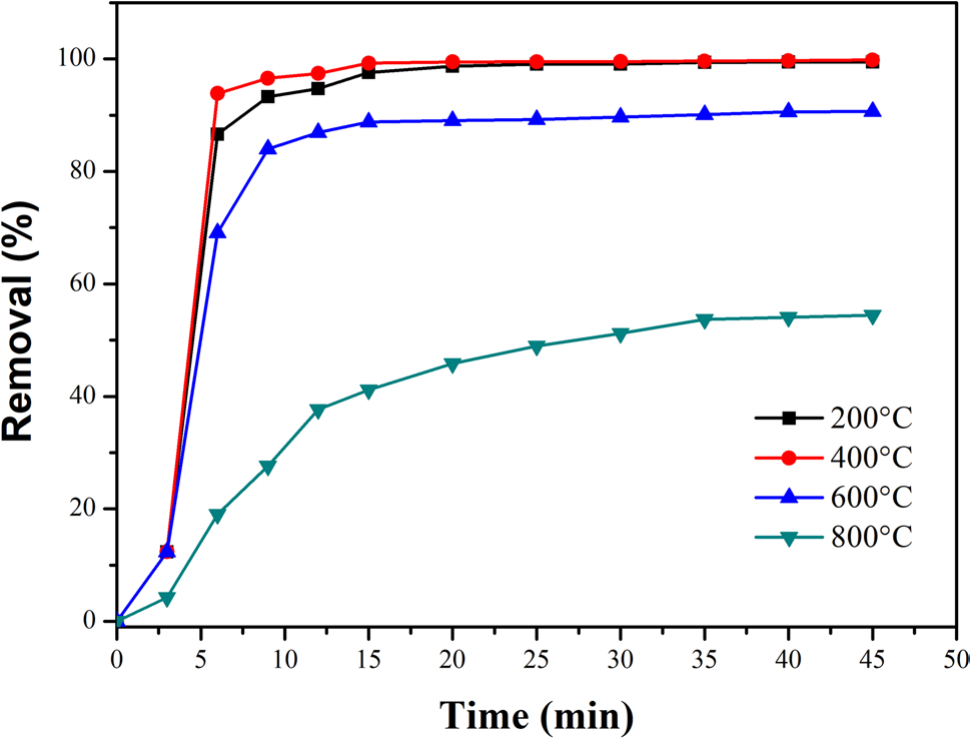
MG removal by adsorption using the annealed CaO-Fe_3_O_4_ composites varying the temperature from 200 to 800 °C. 10 mg of adsorbent and 100 mL of MG (100 ppm) solution were used

The MG removal by adsorption was evaluated using 10 mg of CaO-Fe₃O₄ composites (see Fig. 15) in 100 mL of a 100-ppm MG solution. The CaO-Fe_3_O_4_ composites annealed at 200 and 400 °C exhibited a notable increase in MG removal percentage within the first 15 min, followed by the formation of a pseudo-plateau, indicating equilibrium was reached. The sample annealed at 400 °C demonstrated the highest dye removal efficiency, achieving 99.6% after 45 min of adsorption. This high removal efficiency is due to the composite’s large surface area and the synergistic effects between the CaO and Ca(OH)_2_ phases. A similar trend was observed for the CaO-Fe_3_O_4_ composite annealed at 600 °C, though with slightly reduced efficiency compared to the 400 °C sample, likely due to a decrease in surface area. Conversely, the sample annealed at 800 °C showed a marked decline in removal efficiency, which can be attributed to a further reduction in surface area and the predominant presence of the CaO phase.

**Fig. 16:**
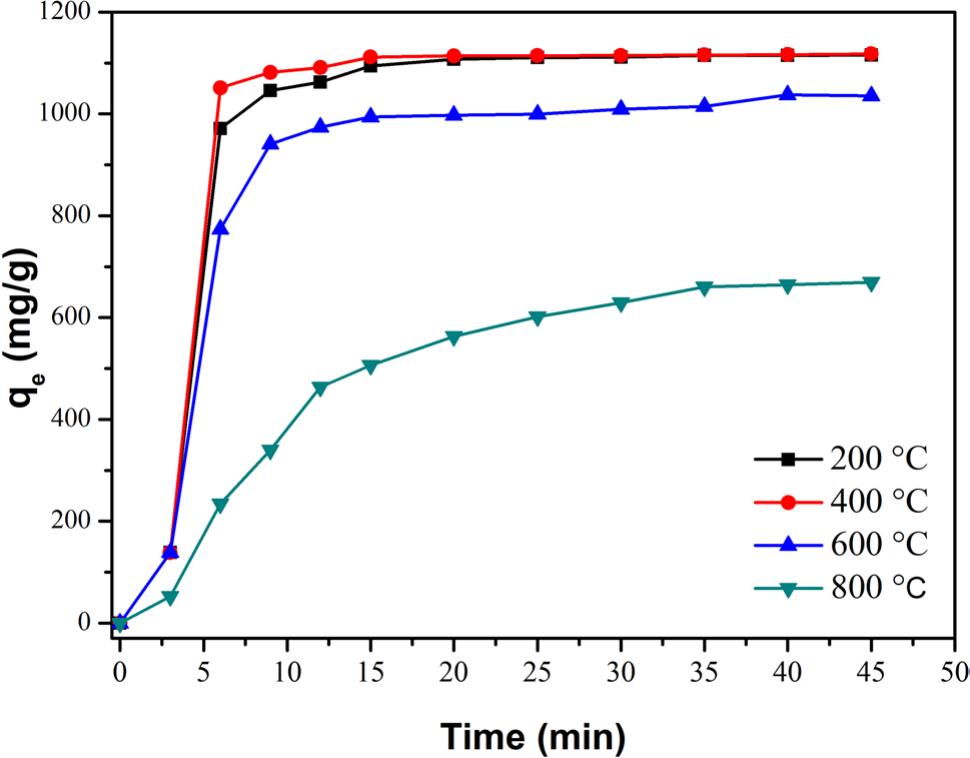
Variation of equilibrium adsorption capacity of MG using the annealed CaO-Fe_3_O_4_ composites varying the temperature from 200 to 800 °C. 10 mg of adsorbent and 100 mL of MG (100 ppm) solution were used

The *q*_e_ variation of MG over time is depicted in Fig. 16, revealing fast adsorption, except for the sample annealed at 800 °C. During the first 15 min, *q*_e_ increased rapidly, reflecting the availability of numerous active sites at the beginning of the process. For the samples annealed at 200, 400, and 600 °C, *q*_e_ remains nearly constant after 15 min, which can be attributed to the dye molecules coating the interior surface of the adsorbent. This saturation of binding sites ultimately leads to equilibrium. The adsorbent’s characteristics significantly affect the time required to reach equilibrium. Notably, the sample annealed at 800 °C requires approximately 35 min to achieve equilibrium, likely due to its larger particle size, reduced surface area, and predominant CaO phase.

The adsorption kinetics describes the rate at which the adsorbate adheres to the adsorbent and also determines the equilibrium time. In this study, typical first-order kinetics was initially applied to the MG adsorption process. However, the logarithmic change over time did not reveal a clear trend or provide definitive information regarding the adsorption process. To better understand the mechanisms involved, both the pseudo-first-order and pseudo-second-order models were employed to analyze the dye adsorption kinetic curves in CaO-Fe_3_O_4_ composites. The fitting curves of the pseudo-first-order model and the corresponding parameters are shown in Fig. 1S (support information). Although the *R*^2^ values were reasonably high in some cases, the calculated *q*_e_ values obtained from this kinetic model are excessively high compared to the experimental *q*_e_ values (see Table 5). This discrepancy suggests that the adsorption process does not align with Lagergren’s pseudo-first-order adsorption rate model.

**Table 5:** Kinetic parameters of MG adsorption by CaO-Fe_3_O_4_ composites obtained from the analysis of adsorption kinetics using the pseudo-first-order and pseudo-second-order models

Sample	*q*_e,experimental_ (mg/g)	Pseudo-first-order			Pseudo-second-order		
		*q*_e, calculated_	*K*_1_ (min^−1^)	*R*^2^	*q*_*e*calculated_	*K*_2_ (g⋅mg ^−1^⋅min^−1^)	*R*^2^
200 °C	1107.58	1312.908	− 0.7082	0.950	1111.11	0.0012	0.999
400 °C	1111.72	1305.968	− 0.8777	0.872	1111.11	0.0027	0.999
600 °C	999.54	1408.380	− 0.7777	0.974	1098.90	0.0004	0.993
800 °C	660.01	757.103	− 0.2444	0.994	833.33	0.0001	0.991

Then, the pseudo-second-order kinetic model was employed, which is particularly useful in cases of chemisorption, where chemical interactions play a significant role. Furthermore, this model offers a more accurate representation of adsorption kinetics than the pseudo-first-order model, as it incorporates both the adsorption capacity and the amount of adsorbate adsorbed into its kinetic equation. The pseudo-second-order reaction kinetics are defined by Eq. 15 (Ho and McKay 1998), where *q*_*t*_ and *q*_e_ are the amounts of MG adsorbed on the CaO-Fe_3_O_4_ adsorbent at time *t* and equilibrium, respectively. *K*_2_ is the pseudo-second-order rate constant; *K*_2_ and *q*_e_ were calculated from the intersection and slope of the plot (*t/q*_*t*_) vs time.

15 $$\frac{t}{{q}_{t}}=\frac{1}{{K}_{2}{q}_{e}^{2}}+\frac{t}{{q}_{e}}$$

**Fig. 17:**
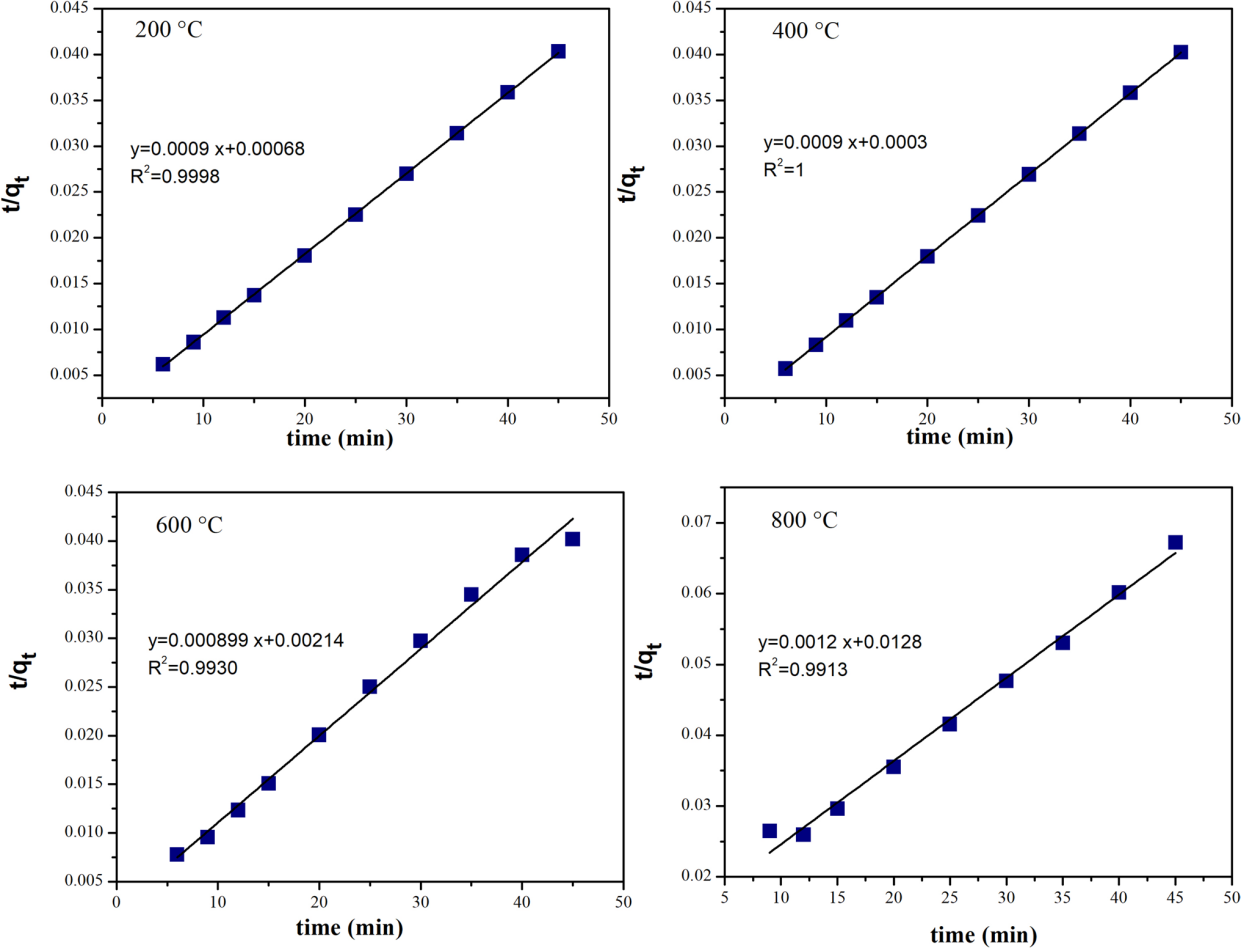
Pseudo-second-order adsorption kinetics of MG on the absorbent CaO-Fe_3_O_4_ varying the annealing temperature from 200 to 800 °C

With strong correlation coefficients, it is clear from Fig. 17 that the pseudo-second-order equation fits the data better than the pseudo-first-order equation (see Table 5). Moreover, the estimated *q*_e_ values closely align with the experimental values for MG, suggesting that the adsorption process involves a chemisorption phenomenon (López-Luna et al. 2019). The dye adsorption likely takes place through exchange reactions on the surface until the functional sites are completely occupied. Following this, the dye molecules may diffuse into the structure, where they interact and/or engage in subsequent reactions (Crini et al. 2007).

Additionally, the adsorption isotherms were analyzed using the Freundlich and Langmuir models to distinguish whether the adsorption process is governed by chemisorption or physisorption (Allen et al. 2004). The Freundlich model, which applies to heterogeneous surfaces, is particularly relevant for multisite or multilayer adsorption. This model employs an exponential equation that suggests non-uniformity in the adsorbent surface, leading to adsorption occurring across multiple layers. As the adsorbate concentration increases, the amount of adsorbate also enhances on the surface.

The heterogeneity factor in the Freundlich isotherm, represented by the parameter 1/*n*, indicates the inhomogeneity of the adsorbent’s surface and characterizes the variability in adsorption site affinity (Cano et al. 2023). The Freundlich isotherm is expressed in both nonlinear and linear forms, as described by Eqs. 16 and 17, respectively. The *k*_f_ and *n* constants are isothermal parameters indicative of the adsorption capacity and intensity of the adsorption driving force magnitude, respectively. The *C*_e_ (mg/L) and *q*_*e*_ (mg/g) parameters are the concentrations in the liquid and solid phases of the adsorbate. To calculate the *k*_f_ and 1/*n* constants, the plot *Log*(*q*_*e*_) *vs. Log*(C_e_) was employed (Fig. 2S support information) using Eq. 16, and their values were estimated from the *y*-intercept and the slope.

16 $${q}_{e}={k}_{f}{{C}_{e}}^{1/n}$$

17 $$Log{q}_{e}=Log{k}_{f}+Log{C}_{e}$$

An *n* value of less than 1 in Freundlich isotherms indicates a more linear and less favorable adsorption, reflecting a lower adsorption capacity or affinity of the adsorbent towards the adsorbate than higher *n* values. Notably, the sample annealed at 400 °C showed the highest *n* value of 2.64, indicating a high adsorption capacity to the MG dye. In comparison, the sample annealed at 800 °C demonstrates an *n* value of 0.58, indicating less favorable adsorption and a reduced adsorption capacity of the adsorbent towards the adsorbate. Although this model shows high correlation values (see Table 6), it does not accurately represent the malachite green adsorption process on the absorbent.

**Table 6:** Adsorption isotherms for the MG adsorption by CaO-Fe_3_O_4_ composite varying the annealing temperature

Sample	Adsorption isotherms						
	Freundlich			Langmuir			
	*K*_f_	*n*	*R*^2^	*Q*_max_ (mg/g)	*K*_L_ (L/mg)	*R*_L_	*R*^2^
200 °C	0.65	2.60	0.951	136.98	0.3310	0.026	0.982
400 °C	0.63	2.64	0.981	136.98	0.6822	0.022	0.996
600 °C	25.11	1.29	0.858	128.20	0.0813	0.098	0.949
800 °C	197.94	0.58	0.951	142.85	0.0210	0.278	0.918

The Langmuir isotherm is a key model used to describe adsorption on a monolayer of a perfectly homogeneous surface, where the number of identical adsorption sites is finite and molecular interactions are minimal. Equation 18 (Ullah et al. 2017) represents the Langmuir equation in its linear form. In this equation, the parameters *Q*_max_ and *K*_L_ are Langmuir constants.* Q*_max_ (mg/g) is the maximum adsorption capacity, and *K*_L_ (L/mg) is the adsorption speed related to the affinity of the adsorbent and adsorbate, *C*_e_ (mg/L) is the equilibrium concentration, and *q*_*e*_ is the amount of adsorbed material per unit mass of adsorbent (mg/g). The values of *Q*_max_ and *K*_L_ can be calculated by plotting *C*_e_*/q*_e_ vs. *C*_e_. The Langmuir isotherm can be analyzed in terms of the dimensionless constant, *R*_L_, given by Eq. 19 (Hameed and El-Khaiary 2008).

18 $$\frac{{C}_{\text{e}}}{{q}_{\text{e}}}=\frac{1}{{Q}_{\text{max}}{K}_{\text{L}}}+\frac{1}{{Q}_{\text{max}}}{C}_{\text{e}}$$

19 $${R}_{\text{L}}=\frac{1}{1+{C}_{o}{K}_{\text{L}}}$$

where *C*_0_ is the initial MG concentration and the parameter *R*_*L*_ indicates the type of adsorption, being favorable (0 < *R*_L_ < 1), unfavorable (*R*_L_ > 1), linear (*R*_L_ = 1), and irreversible (*R*_L_ = 0).

**Fig. 18:**
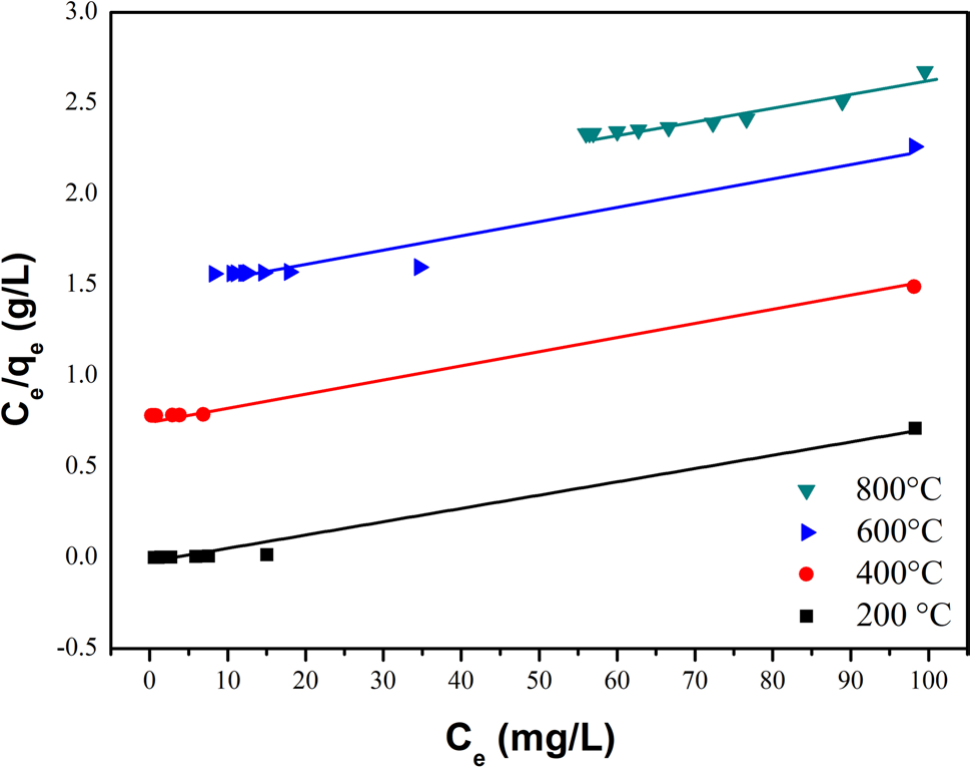
Adsorption Langmuir isotherm study of MG adsorption using CaO-Fe_3_O_4_ varying the annealing temperature from 200 to 800 °C

The Langmuir constants and the *R*_L_ parameter are reported in Table 6. All the composites exhibit a good linear correlation coefficient between 0.9182 and 0.9962 (see Fig. 18), while *R*_L_ values are below 1, indicating favorable adsorption since it implies that the adsorbate is easily adsorbed on the surface of the adsorbent. The saturation capacity of the monolayer for the composite treated at 400 °C was 136.98 g/mg. This measurement indicates the maximum adsorption capacity the surface can reach before completely becoming saturated. Higher *K*_L_ values (such as 0.6822 for the sample treated at 400 °C) indicate a greater affinity and adsorption capacity of the adsorbate towards the solid surface (Vasanth Kumar et al. 2005).

Adsorption capacity is crucial for evaluating the efficiency of materials in removing dyes such as MG. The hybrid CaO-Fe₃O₄ material, developed from eggshells through mechanical ball milling with Fe₃O₄ synthesized at 400 °C, achieved a maximum *q*_e_ value of 1111.11 mg/g with a removal efficiency of 99.6% in 45 min. In comparison, micromesoporous carbon activated with NaOH achieved a higher adsorption capacity (i.e., 1300 mg/g), but its synthesis required intensive chemical reagents (furfural, hydroquinone, urotropine) and multiple stages of thermal and chemical activation, increasing its complexity and cost (Memetova et al. 2024). In contrast, the CaO-Fe₃O₄ composite utilizes waste materials in a more sustainable process that is less chemically intensive. Additionally, the magnetic nature of Fe_3_O_4_ facilitates recovery after the adsorption process. A noteworthy advantage of this material is its significantly higher adsorption capacity compared to other adsorbents with similar base components, such as Ca(OH)₂ or Fe₃O₄-based hybrid materials, which did not achieve high adsorption efficiencies, as shown in Table 7. However, it is important to highlight that some materials exhibit even higher MG removal capacities, though these were synthesized through complex chemical processes and commercial, high-purity, and sophisticated chemical reagents. These characteristics make CaO-Fe₃O₄ a scalable and environmentally friendly alternative for industrial applications, offering an optimal balance of performance, cost, and sustainability while overcoming the limitations of materials with similar compositions.

**Table 7:** MG adsorption reports on different adsorbents

Material	Synthesis method	Dosage catalyst mg/mL	Dye (ppm)	Adsorption capacity *q*_e_ (mg/g)	Removal (%)	Time (min)	Ref
Ca(OH)_2_	Mashing/activation	10/100	10 to 100	14	*	20	Chowdhury and Saha (2011)
Fe_3_O_4_/SC	Ultrasonic-assisted synthesis	59/100	10 to 100	41.66	89.22	59.86	Bonyadi et al. (2022)
Activated carbon with iron oxide	Sol–gel	30/20	100	700	*	45	Lincold et al. (2024)
Alg-Fe_3_O_4_	Co-precipitation	100/50	10	16.21	82	20	Mohammadi et al. (2014)
CaO-ZnFe_2_O_4_	Ball-milling/combustion	5/250	100	2148	49.56	55	Reyes-Vallejo et al. (2024b)
Mesoporous carbon	Alkaline activation (NaOH)	*	500	1300	*	30	Memetova et al. (2024)
Activated biochar	Chemical activation	100/100	10	992.72	88.4	30	Cano et al. (2025)
TpStb-SO₃Na	Schiff-base condensation	2.5/10	2000	5857	*	48 (h)	Li et al. (2023)
Porous nanosilica microspheres	Gel-sol and etching	10/50	2000	3831	*	24 (h)	Chen et al. (2025)
Carbon/zeolite (CZCM)	Pyrolysis	0.5/5	1000	9705	97.05	24 (h)	Shi et al. (2025)
CaO/Fe_3_O_4_	Ball milling/annealing	10/100	100	1111.11	99.6	45	**This work**

^*^Not mentioned in the report

**Fig. 19:**
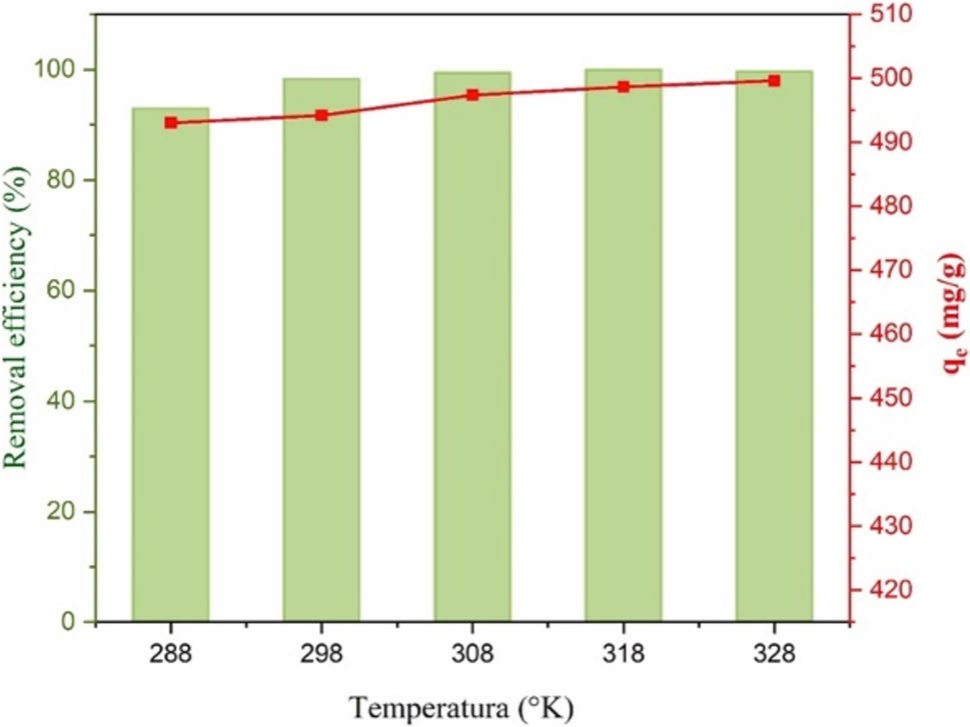
Effect of temperature on the adsorption of MG dye on CaO-Fe_3_O_4_ composite annealed at 400 °C. For the test, 50 ppm of MG, 12 min of contact, and 10 mg of adsorbent were used

In addition, to understand how temperature influences the adsorption capacity at equilibrium (*q*_*t*_) and the removal percentage of MG on the composite annealed at 400°C (see Fig. 19), a temperature sweep ranging from 288 to 328 K (i.e., 15–55 °C) was conducted over 12 min without pH modifications. The MG solution was heated or cooled to the desired temperature. A cooling system was employed to achieve a temperature of 288 K (15 °C), while a heating plate was used for higher temperatures. The solution’s temperature was continuously monitored throughout the experiment to ensure it remained at the target temperature for each study point. The results showed that the MG removal percentage and the amount of adsorbed MG increased with rising temperature, indicating an endothermic process (Robati et al. 2016). This indicates that higher temperatures generally facilitate the interaction between the adsorbate and the adsorbent, likely due to the greater mobility of adsorbate molecules, thereby enhancing the adsorption process.

The thermodynamic parameters offer important insights into the nature and viability of an adsorption process. These include the Gibbs free energy (*∆G°*), enthalpy change (*ΔH°*), and entropy change (*ΔS°*). From a thermodynamic perspective, these parameters make it possible to ascertain spontaneity, whether energy is absorbed or released, and the change in entropy during the adsorption process. Based on the variation of the thermodynamic equilibrium constant (*K*_*d*_) with temperature change, changes in the thermodynamic parameters of adsorption, such as *∆G°*, *ΔH°*, and *ΔS°*, were estimated using Eq. 20.

20 $${\Delta G}^{0}=-RTln{K}_{d}$$

where *T* is the absolute temperature (*K*) and *R* is the gas constant (8.3145 × 10^−3^ kJ/mol K). *K*_*d*_ is calculated from experimental data by using Eq. 21 (Rosa 2008).

21 $${K}_{d}=\frac{{C}_{se}}{{C}_{e}}$$

where *C*_e_ is the dye concentration in solution (mg/L) and *C*_se_ is its equilibrium concentration in the solid phase (mg/L). The changes in enthalpy and entropy can be obtained by using Eqs. 22 and 23, respectively.

22 $$ln{K}_{d}=\frac{{\Delta S}^{0}}{R}+ \frac{{\Delta H}^{0}}{RT}$$

23 $$\Delta {G}^{0}=\Delta {H}^{0}-T\Delta {S}^{0}$$

**Fig. 20:**
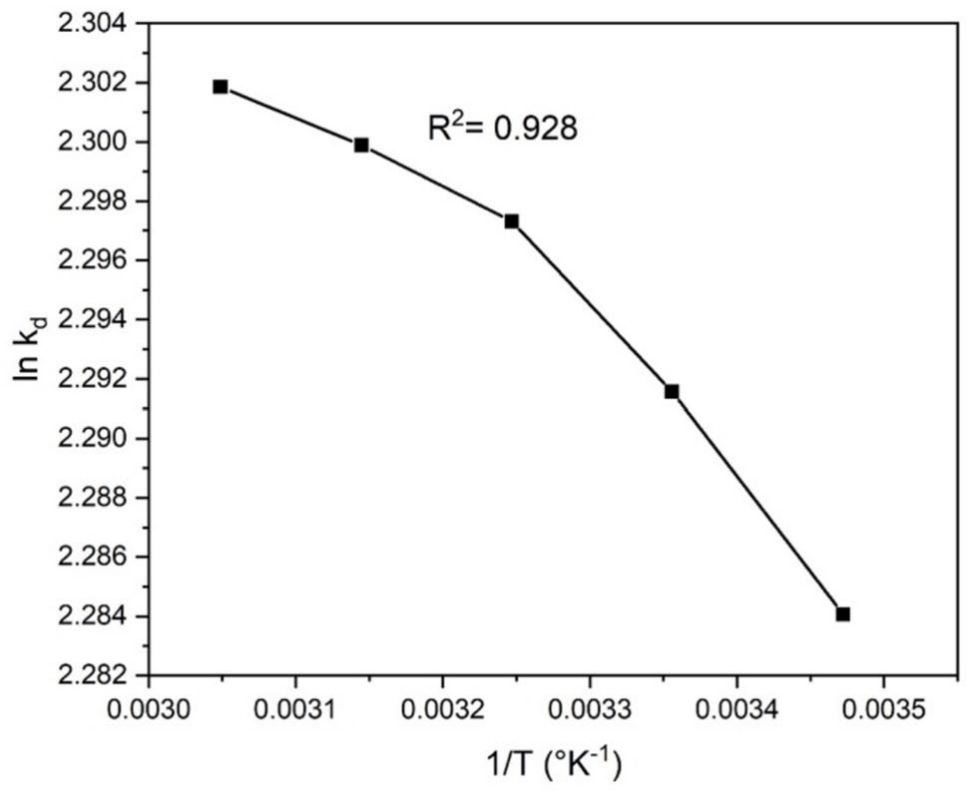
Plot of the kinetic constant (ln*k*_d_) vs. temperature (1/*T*). The thermodynamic parameters in Table 8 are determined from this graph

The slope and the intercept obtained from the ln *K*_*d*_ vs. *1/ T* plot, using Eq. 22, were used to obtain the values of ${\Delta H}^{0}$ and ${\Delta S}^{0}$ (Fig. 20).

Table 8 shows the thermodynamic parameter values. The negative *∆G°* values indicate that adsorption is thermodynamically feasible across all measured temperatures, suggesting a spontaneous process. As temperature increases, the *∆G°* values are more negative, indicating that adsorption is more spontaneous (Al-Asadi et al. 2023). The positive *∆H°* value implies that the adsorption process absorbs heat from the surroundings, indicating an endothermic process (Liu et al. 2019). The positive ${\Delta S}^{0}$ value suggests increased randomness or disorder at the adsorbent-solution interface during adsorption (Gebreslassie 2020). Therefore, the combination of positive enthalpy and entropy influences the adsorption process, involving strong chemical interactions, such as the formation of chemical bonds between the dye molecules and the adsorbent. At the same time, the increase in molecular disorder contributes to the spontaneity of the process.

**Table 8:** Thermodynamic parameters values for the adsorption of MG on CaO-Fe_3_O_4_ at different temperatures

*Temperature*		*Thermodynamic parameters*			
°K	°C	${K}_{d}$	∆${G}^{0}$(KJ/mol)	$\Delta {H}^{0}$(KJ/mol)	$\Delta {S}^{0}$(J/mol °K)
*288*	15	9.81	− 5.469	0.349	20.28
*308*	35	9.89	− 5.678		
*318*	45	9.94	− 5.883		
*328*	55	9.97	− 6.081		

## Conclusions

This study developed an innovative and sustainable approach for synthesizing CaO-Fe₃O₄ composites using waste-derived materials and an eco-friendly synthesis route for the efficient removal of water pollutants. The composites were subjected to thermal treatments to analyze their effect on structural properties and dye removal efficiency. The sample treated at 400 °C exhibited unique characteristics, such as a high surface area, lower bandgap energy, and enhanced crystallinity, which significantly improved its performance in both adsorption and photocatalysis. This material achieved complete degradation of MB at 10 ppm within 30 min and at 50 ppm within 90 min, as well as 99.6% removal of MG through adsorption, reaching maximum adsorption capacity in just 45 min.

The photocatalytic degradation of MB was primarily governed by superoxide radicals and holes, as evidenced by radical trapping experiments. On the other hand, the adsorption of MG followed pseudo-second-order kinetics and the Langmuir isotherm model, indicating a chemisorption mechanism and monolayer adsorption. The endothermic nature of the adsorption process was confirmed by the increase in MG removal efficiency with rising temperature, further supported by positive enthalpy values.

Furthermore, the combination of Fe₃O₄ and CaO enhanced the material’s stability and imparted magnetic properties, facilitating its recovery and reinforcing its applicability in large-scale processes aligned with circular economy principles. A key aspect of this work is the use of ball milling as a synthesis method, an economical, scalable, and easily implementable technique that enables the production of homogeneous materials without the need for toxic reagents or extreme temperature and pressure conditions.

To the best of our knowledge, no previous studies have reported the use of CaO-Fe₃O₄ composites for the removal of these specific dyes, highlighting the novelty and relevance of this work in exploring new materials for wastewater treatment. However, one of the main limitations is the need to evaluate the material’s stability and performance under varying environmental conditions, which represents an opportunity for future research. Overall, this waste-derived composite offers a cost-effective, sustainable, and highly efficient solution for water treatment, contributing significantly to global environmental sustainability goals.

## Supplementary Information

Below is the link to the electronic supplementary material.

Supplementary file1 (DOCX 232 KB)

## Data Availability

The datasets generated during and/or analyzed during the current study are available from the corresponding author on reasonable request.
