# Supplementary material for: Green synthesis of CaO-Fe₃O₄ composites for photocatalytic degradation and adsorption of synthetic dyes
Source: Environ Sci Pollut Res Int. 2025 Mar 31;32(15):9901–25. doi: 10.1007/s11356-025-36310-w (PMC11991969; doi:10.1007/s11356-025-36310-w)
Supplement: Supplementary file 1 — Supplementary file1 (DOCX 232 KB) [file 11356_2025_36310_MOESM1_ESM.docx]

**Support information of**

Green Synthesis of CaO-Fe₃O₄ Composites for Photocatalytic Degradation and Adsorption of Synthetic Dyes

Odín Reyes-Vallejo^1*^, Rocío Magdalena Sánchez-Albores^2^_,_ José Escorcia-García^3^, Abumale Cruz-Salomón^2^, Pascual Bartolo-Pérez^4^, Ashok Adhikari^5^, Maritza del Carmen Hernández-Cruz^2^, Héctor Hiram Torres-Ventura^2^, Héctor Armando Esquinca-Avilés^2^

^1^Sección de Electrónica de Estado Sólido-Ingeniería eléctrica (SEES), CINVESTAV- IPN, San Pedro Zacatenco, Ciudad de México, 07360, México.

^2^Escuela de Ciencias Químicas, Universidad Autónoma de Chiapas (UNACH), Ciudad de Ocozocoautla de Espinosa 29140, Chiapas, México.

^3^CONAHCYT-CINVESTAV del IPN, Unidad Saltillo, Ciudad de Ramos Arizpe 25900, Coahuila, México.

^4^Departamento de Física Aplicada, Centro de Investigación y de Estudios Avanzados del Instituto Politécnico Nacional-Unidad Mérida, Ciudad de Mérida 97310, Yucatán, México.

^5^Departamento de Materia Condensada, Instituto de Física-UNAM, Coyoacán 04510, Ciudad de México, México.

*Corresponding author: odin.reyes.v@cinvestav.mx

The pseudo-first-order kinetic model assumes that the absorption rate of a solute over time is proportional to the difference between the saturation concentration and the amount of solids’ absorption over time. In addition, it is noted that this equation is used to distinguish a kinetic based on the adsorption capacity of a solid from a kinetic based on the concentration of a solution.

The pseudo-first-order model is commonly used to describe the initial phase of an adsorption process. This model is expressed by equation 1s:

$\frac{dq_{t}}{dt}=K_{1}(q_{e}-q_{t})$ (1s)

Where K_1_(𝑚𝑖𝑛^−1^) is the rate constant, 𝑞_𝑡_(𝑚𝑔/g) is the amount of adsorbate adsorbed over time. By integrating and making the limit condition, 𝑡 = 0 and 𝑞_𝑡_ = 0, 𝑞_𝑒_ = 𝑞_𝑡_ and 𝑡 = 𝑡, gives the equation 2s (Robati *et al*., 2016):

$log\left( q_{e}-q_{t} \right)=log\left( q_{e} \right)-\frac{k_{1}}{2.303}t$ (2s)

where q_t_ and q_e_ are the amounts of MG adsorbed on the CaO/Fe_3_O_4_ adsorbent at time *t* and equilibrium, respectively. The rate constant K_1_ is determined by plotting the values of log(q_e_ − q_t_) vs *t* (Figure 1S), whereby K_1_ and q_e_ are calculated from the slope and intercept, respectively.

The linear plots of log(q_e_ - q_t_) *vs t* (only for the initial adsorption period - 15 minutes) show that the dye adsorption process follows first-order kinetics. However, although the R^2^ values are reasonably high, in some cases, the calculated q_e_ values obtained from this kinetic model are too high compared to the experimental q_e_ values (Table 5). This analysis indicates that the adsorption process does not fit Lagergren's pseudo-first-order adsorption rate expression.

In the pseudo-first-order kinetics, the sample annealed at 400 °C has a deviation from the straight line in the first 6 min of sorption (Figure 1S). This behavior was attributed to the rapid initial adsorption of MG on CaO/Fe_3_O_4_.


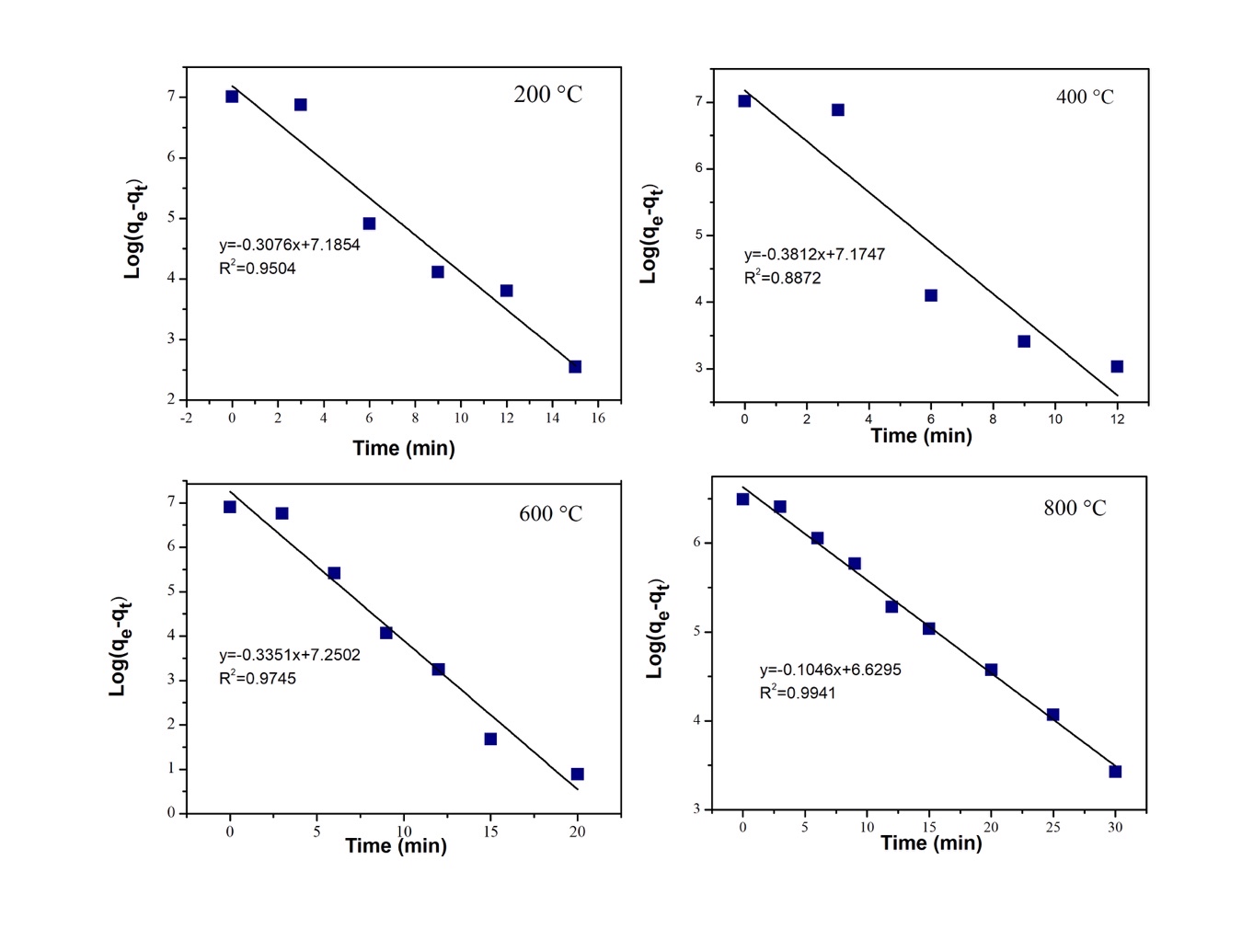


***Fig. 1S*** *Pseudo-first-order adsorption kinetics of MG on the absorbent CaO/Fe_3_O_4_ varying the annealing temperature from 200 to 800 ºC.*


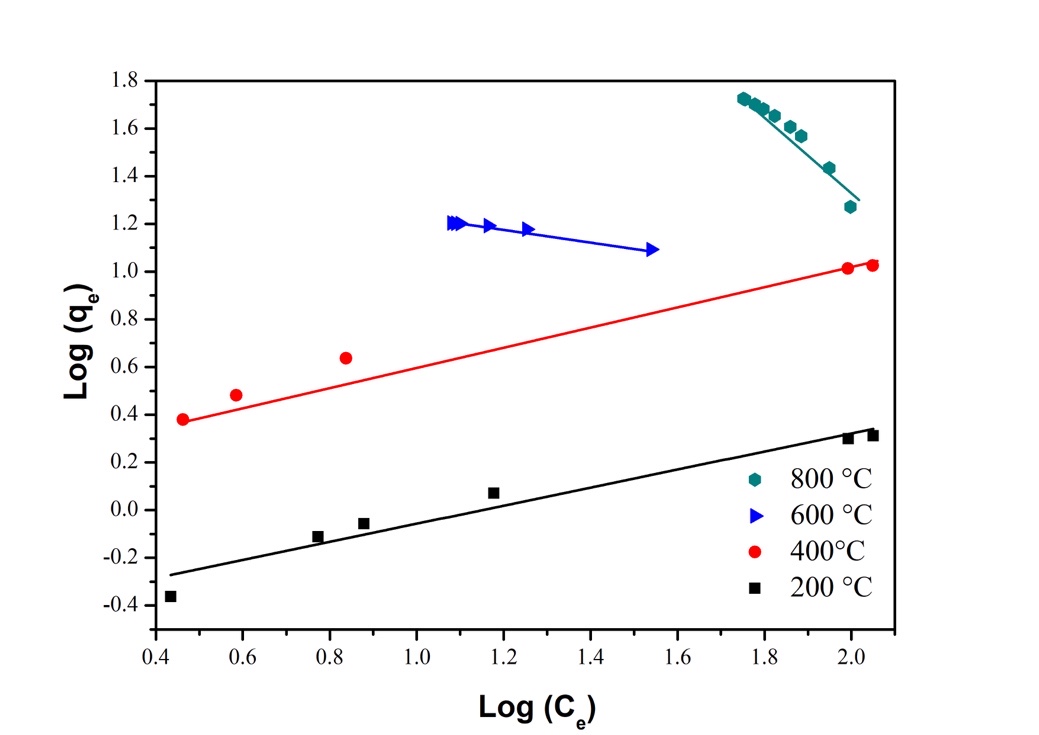


***Fig. 2S*** *Freundlich isotherm study of MG adsorption using CaO/Fe_3_O_4_ varying the annealing temperature from 200 to 800 ºC.*

**References:**

Robati, D., Rajabi, M., Moradi, O., Najafi, F., Tyagi, I., Agarwal, S., & Gupta, V. K. (2016). Kinetics and thermodynamics of malachite green dye adsorption from aqueous solutions on graphene oxide and reduced graphene oxide. *Journal of Molecular Liquids*, *214*. https://doi.org/10.1016/j.molliq.2015.12.073
